# Ultra-strong nonlinear optical processes and trigonal warping in MoS_2_ layers

**DOI:** 10.1038/s41467-017-00749-4

**Published:** 2017-10-12

**Authors:** Antti Säynätjoki, Lasse Karvonen, Habib Rostami, Anton Autere, Soroush Mehravar, Antonio Lombardo, Robert A. Norwood, Tawfique Hasan, Nasser Peyghambarian, Harri Lipsanen, Khanh Kieu, Andrea C. Ferrari, Marco Polini, Zhipei Sun

**Affiliations:** 1 Department of Electronics and Nanoengineering, Aalto University, Tietotie 3, FI-02150 Espoo, Finland; 2 Institute of Photonics, University of Eastern Finland, Yliopistokatu 7, FI-80100 Joensuu, Finland; 3 Istituto Italiano di Tecnologia, Graphene Labs, Via Morego 30, I-16163 Genova, Italy; 4 College of Optical Sciences, University of Arizona, 1630 E University Blvd, Tucson, AZ 85721 USA; 5 Cambridge Graphene Centre, University of Cambridge, Cambridge, CB3 0FA UK

## Abstract

Nonlinear optical processes, such as harmonic generation, are of great interest for various applications, e.g., microscopy, therapy, and frequency conversion. However, high-order harmonic conversion is typically much less efficient than low-order, due to the weak intrinsic response of the higher-order nonlinear processes. Here we report ultra-strong optical nonlinearities in monolayer MoS_2_ (1L-MoS_2_): the third harmonic is 30 times stronger than the second, and the fourth is comparable to the second. The third harmonic generation efficiency for 1L-MoS_2_ is approximately three times higher than that for graphene, which was reported to have a large *χ* ^(3)^. We explain this by calculating the nonlinear response functions of 1L-MoS_2_ with a continuum-model Hamiltonian and quantum mechanical diagrammatic perturbation theory, highlighting the role of trigonal warping. A similar effect is expected in all other transition-metal dichalcogenides. Our results pave the way for efficient harmonic generation based on layered materials for applications such as microscopy and imaging.

## Abstract (web summary)

Harmonic generation is a nonlinear optical process occurring in a variety of materials; the higher orders generation is generally less efficient than lower orders. Here, the authors report that the third-harmonic is thirty times stronger than the second-harmonic in monolayer MoS2.

## Introduction

Nonlinear optical phenomena can generate high-energy photons by converting *n* = 2, 3, 4,… low-energy photons into one high-energy photon. These are usually referred to as second-, third-, and fourth-harmonic generation (SHG, THG, and FHG)^1^. Due to different selection rules^1, 2^, harmonic processes are distinct from optically pumped laser phenomena (e.g., optically pumped amplification^3^), and other typical single-photon processes (e.g., single-photon excited photoluminescence^1^), in which the energy of the generated photons is smaller than the pump photons. Multiphoton harmonic processes have been widely exploited for various applications (e.g., all-optical signal processing in telecommunications^1, 4^, medicine^5^, and data storage^6^), as well as to study various transitions forbidden under low-energy single-photon excitation^5, 6^. The physical origin of these processes is the nonlinear polarization induced by an electromagnetic field **E**. This gives rise to higher harmonic components, the *n*-th harmonic component amplitude being proportional^1^ to |**E**|^*n*^. Quantum mechanically, higher-harmonic generation involves the annihilation of *n* pump photons and generation of a photon with *n* times the pump energy. Because an *n*-th order nonlinear optical process requires *n* photons to be present simultaneously, the probability of higher-order processes is lower than that of lower order^1^. Thus, higher-order processes are typically weaker and require higher pump intensities^7, 8^.

Graphene and related materials are at the center of an ever-increasing research effort due to their unique and complementary properties, making them appealing for a wide range of photonic and optoelectronic applications^9–11^. Among these, semiconducting transition-metal dichalcogenides (TMDs) are of particular interest due to their direct bandgap when in monolayer (1L) form^12^, leading to an increase in luminescence by a few orders of magnitude compared with the bulk material^12, 13^. 1L-MoS_2_ has a single layer of Mo atoms sandwiched between two layers of S atoms in a trigonal prismatic lattice. Therefore, in contrast to graphene, it is non-centrosymmetric and belongs to the space group $D_{3{\rm{h}}}^1\!$ ^14^. The lack of spatial inversion symmetry makes 1L-MoS_2_ an interesting material for nonlinear optics, since second-order nonlinear processes are present only in non-centrosymmetric materials^1^. However, when stacked, MoS_2_ layers are arranged mirrored with respect to one another^14^, therefore MoS_2_ with an even number of layers (EN) is centrosymmetric and belongs to the $D_{3{\rm{d}}}^3$ space group^14^, producing no second harmonic (SH) signal. On the other hand, MoS_2_ with odd number of layers (ON) is non-centrosymmetric. SHG from 1L-MoS_2_ was reported by several groups^14–21^.

Here we present combined experimental and theoretical work on nonlinear harmonic generation in 1L and few-layer (FL) MoS_2_. We report strong THG and FHG from 1L-MoS_2_. THG is more than one order of magnitude larger than SHG, while FHG has the same magnitude as SHG. This is surprising, since one normally expects the intensity of nonlinear optical processes to decrease with *n* ^1, 2^, with the SHG intensity much larger than that in THG and FHG, although even-order processes only exist in non-centrosymmetric materials. Our results show that this expectation is wrong in the case of 1L-MoS_2_. At sufficiently low photon frequencies (in our experiments the photon energy of the pump is 0.8 eV), SHG only probes the low-energy band structure of 1L-MoS_2_. This is nearly rotationally invariant^22–29^, but with corrections due to trigonal warping. It is because of these corrections^23, 26, 27^, fully compatible with the $D_{3{\rm{h}}}^1$ space group^1^, but reducing the full rotational symmetry of the low-energy bands to a three-fold rotational symmetry^1^, that a finite amplitude of nonlinear harmonic processes can exist at low photon energies in EN-MoS_2_. The lack of spatial inversion symmetry is a necessary but not sufficient condition for the occurrence of SHG. A purely isotropic band structure gives a vanishing SHG signal^30–33^, despite some terms in the Hamiltonian explicitly breaking inversion symmetry^27, 34–37^. Terms proportional to the *σ*_*z*_ Pauli matrix break inversion symmetry. Breaking the continuous rotational symmetry of isotropic models (e.g., by including trigonal warping) is required to obtain a non-zero second-order response in a two-band system. In hexagonal lattices, trigonal warping is a deviation from purely isotropic bands that emerges as one moves away from the corners **K** and **K**′ of the Brillouin zone^23, 26, 27, 36–38^. Since the lattice has a honeycomb structure, this distortion displays a three-fold rotational symmetry^23, 26, 27, 36–38^. We demonstrate that the observed THG/SHG intensity ratio can be explained by quantum mechanical calculations based on finite-temperature many-body diagrammatic perturbation theory^39^ and low-energy continuum-model Hamiltonians that include trigonal warping^35^. We conclude that, similar to SHG^14–18^, the THG process is sensitive to the number of layers, their symmetry, relative orientation, as well as the elliptical polarization of the excitation light. Similar effects are expected for all other TMDs. This paves the way for the assembly of heterostructures with tailored nonlinear optical properties.

## Results

### Samples

**Fig. 1:**
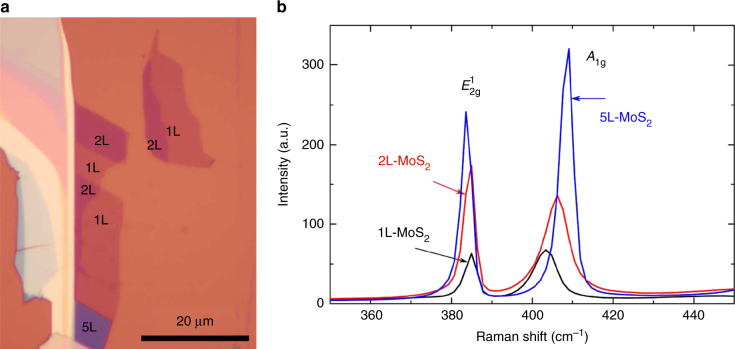
Optical image and Raman spectra of the MoS_2_ flakes. **a** Optical micrograph with single-layer, bilayer, and five-layer areas marked by 1L, 2L, and 5L. **b** Raman spectra of the same sample

MoS_2_ flakes are produced by micromechanical cleavage (MC) of bulk MoS_2_^40^ onto Si + 285 nm SiO_2_. 1L-MoS_2_ and bilayer (2L-MoS_2_) flakes are identified by a combination of optical contrast^41, 42^ and Raman spectroscopy^43^. Raman spectra are acquired by a Renishaw micro-Raman spectrometer equipped with a 600 line/mm grating and coupled with an Ar^+^ ion laser at 514.5 nm. Figure 1a shows the MoS_2_ flakes studied in this work and their Raman signatures. A reference MC graphene sample is also prepared and placed on a similar substrate.

### SHG and THG charcterization

**Fig. 2:**
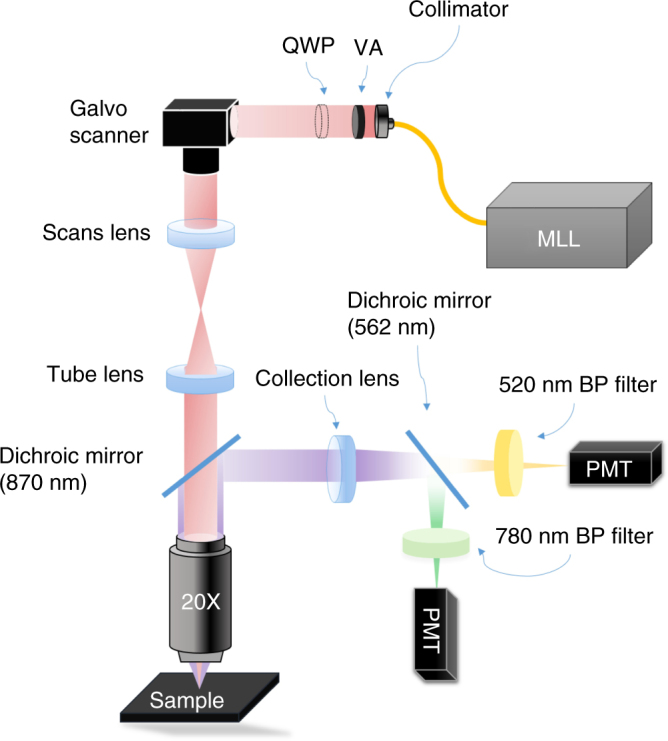
Schematic diagram of multiphoton microscope. MLL, linearly polarized mode-locked fiber laser. VA, variable attenuator. QWP, quarter-wave plate used to study the dependence of SHG and THG on the elliptical polarization of the pump light. BP filter, bandpass filter. PMT, photomultiplier tube

Nonlinear optical measurements are carried out with the set-up shown in Fig. 2 ^44, 45^. As excitation source, we use an erbium-doped mode-locked fiber laser with a 50 MHz repetition rate, maximum average power 60 mW, and pulse duration 150 fs, which yields an estimated pulse peak power of ~8 kW^46^. The laser beam is scanned with a galvo mirror and focused on the sample using a microscope objective. The back-scattered second and third harmonic signals are split into different branches using a dichroic mirror and then detected using photomultiplier tubes (PMTs). For two-channel detection, the light is split into two PMTs using a dichroic mirror with 562 nm cutoff. After the dichroic mirror, the detected wavelength range can be further refined using bandpass filters. The light can also be directed to a spectrometer (OceanOptics QE Pro-FL). The average power on the sample is kept between 10 and 28 mW with a typical measurement time ~5 μs, which prevents sample damage and enables high signal-to-noise-ratio, even with acquisition time per pixel in the μs range.

**Fig. 3:**
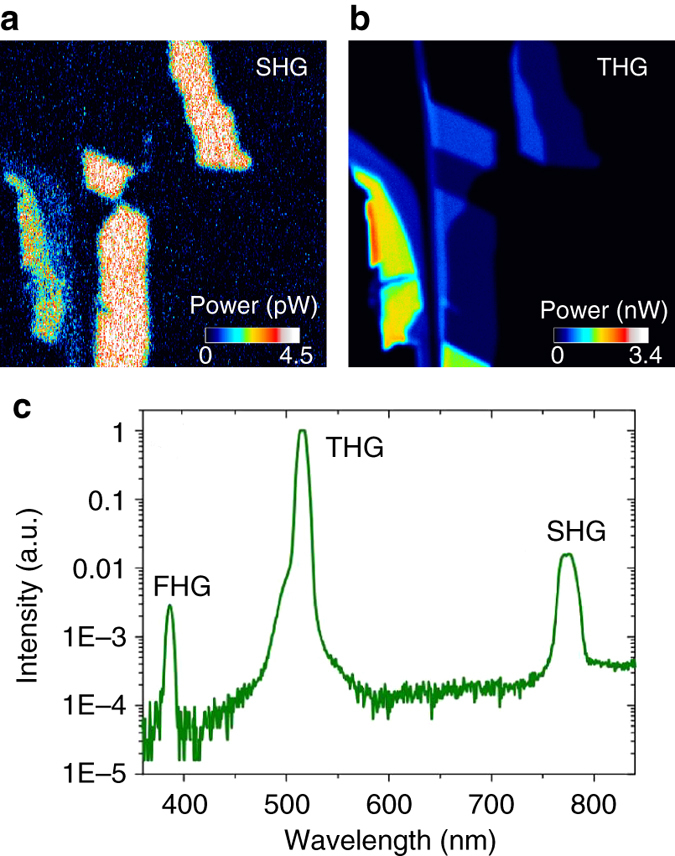
Multiphoton images of MoS_2_ flakes. **a** SHG and **b** THG map of the flakes in Fig. 1a. **c** Optical spectrum of the nonlinear signal from 1L-MoS_2_ with a peak irradiance ~30 GW cm^−2^

SHG and THG images of the MoS_2_ sample are shown in Fig. 3a, b. The SH photon energy is ~1.6 eV, lower than the bandgap of 1L-MoS_2_^12, 13^. This is not unexpected, as harmonic generation can occur when the harmonic energy is below the bandgap^1, 47, 48^. The SHG signal is generated in 1L-MoS_2_, while 2L-MoS_2_ appears dark. As discussed above, the second-order nonlinear response is present in 1L-MoS_2_, which is non-centrosymmetric. However, when stacked to form 2L-MoS_2_, MoS_2_ layers are mirrored^14, 15^. Therefore, EN-MoS_2_ is centrosymmetric^14, 15^, and belongs to the $D_{3{\rm{d}}}^3$ space group^14, 15^, producing no SHG signal. On the other hand, ON-MoS_2_ flakes are non-centrosymmetric^14, 15^.

We note that strong THG is detected compared with SHG, even for 1L-MoS_2_, Fig. 3b. THG was previously reported for a 7L-MoS_2_ flake^18^, but here we see it down to 1L-MoS_2_. Reference ^49^ followed our work^50^ and reported THG and SHG from 1L-MoS_2_, giving effective bulk-like second- and third-order susceptibilities $\chi _{{\rm{eff}}}^{(2)}$ and $\chi _{{\rm{eff}}}^{(3)}$ of 2.9 × 10^−11^ mV^−1^ and 2.4 × 10^−19^ m^2^V^−2^, respectively. However, ref. ^49^ did not provide a detailed explanation of the large THG signal compared to the SHG. Instead it assigned the large THG/SHG ratio to a possible enhancement of THG by the edge of the B exciton. However, refs. ^51, 52^ demonstrated that SHG is enhanced only when the SHG wavelength overlaps the A or B excitons. A similar behavior is expected for THG. Thus, the explanation in ref. ^49^ may not be correct. Reference ^53^ reported high-harmonic (>6th-order) generation in the non-perturbative regime with mid-infrared (IR) excitation (0.3 eV), unlike our THG and FHG results with near-IR excitation (0.8 eV). We do not detect THG from the thickest areas of our flake, with *N* > 30, as in ref. ^18^. The output spectrum in Fig. 3c further confirms that we observe both SHG and THG. Peaks for THG and SHG at 520 and 780 nm can be seen, as well as at 390 nm, corresponding to a four-photon process. This is detected only in 1L-MoS_2_. Its intensity is ~5.5 times lower than SHG, and two orders of magnitude smaller than THG.

**Fig. 4:**
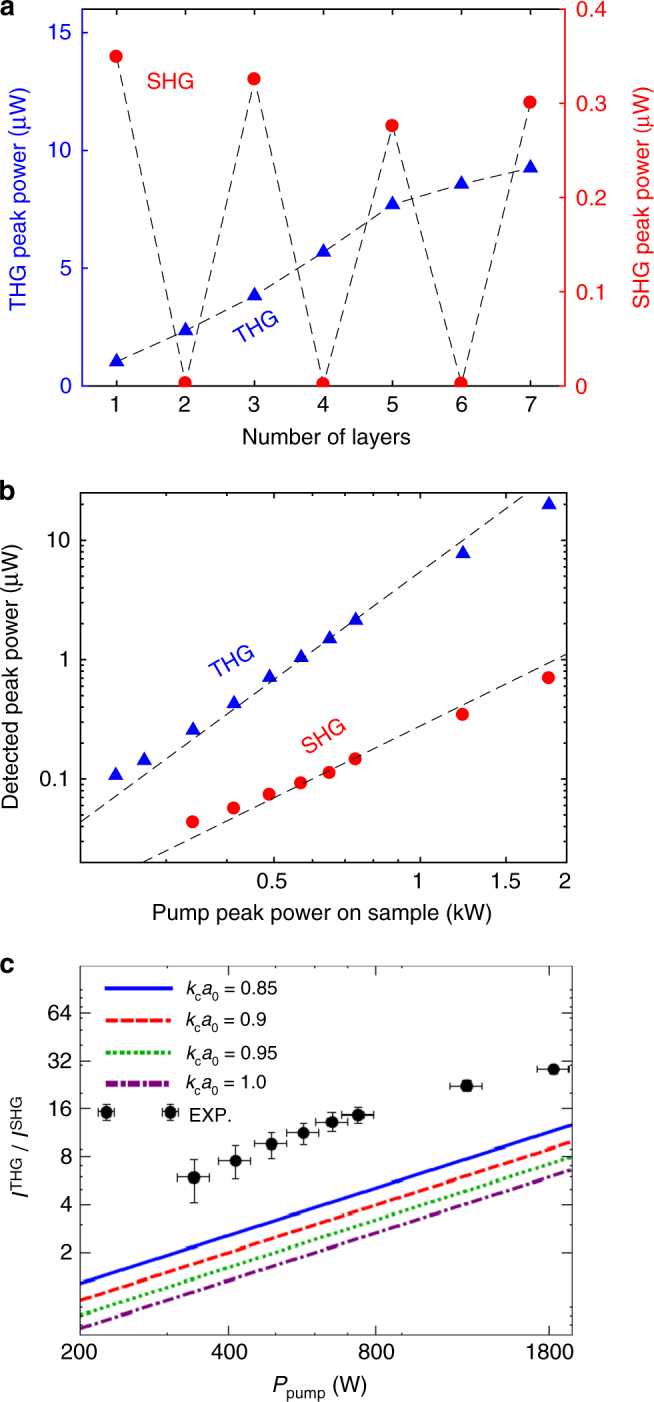
Experimental and theoretical nonlinear optical processes in MoS_2_. **a** SHG and THG intensities as functions of N. **b** Power dependence of SHG and THG in 1L-MoS_2_. **c** Experimental and theoretical THG/SHG irradiance ratio as a function of *P*_pump_. Different theoretical curves refer to different values of the ultra-violet cutoff *k*_c_ (in units of $1{\rm{/}}{a_0} = \sqrt 3 {\rm{/}}a,$ where *a* = 3.16 Å is the lattice constant of 1L-MoS_2_^12^). Black dashed lines in panels **a** and **b** are a guide to the eye. The error bars in **c** account for experimental uncertainties

SHG signals on areas with *N* = 3, 5, 7 have nearly the same intensity as 1L-MoS_2_, Fig. 4a. This contrasts ref. ^14^, where a pump laser at 810 nm was used. We attribute this difference to the fact that photons generated in the second-order nonlinear process in our set-up with a 1560 nm pump have an energy ~1.6 eV (780 nm), below the band gap of 1L-MoS_2_^12^, therefore are not adsorbed, unlike the SHG signal in ref. ^14^.

### Second- and third-order nonlinear susceptibilities

Based on the measured SHG and THG intensities, we can estimate the nonlinear susceptibilities *χ* ^(2)^ and *χ* ^(3)^. *χ* ^(2)^ can be calculated from the measured average powers of the fundamental and SH signals as follows^54^:

1 $$\chi _{\rm{s}}^{(2)} = \sqrt {\frac{{{\epsilon _0}c\lambda _2^4{P_{2\omega }}R{\tau ^2}{{\left( {{n_2} + 1} \right)}^2}{{\left( {{n_1} + 1} \right)}^2}}}{{32N_a^2{\tau _2}{P_{{\rm{pump}}}}\phi }}} ,$$

where *τ* is the pulse width, *P*_pump_ is the average power of the incident fundamental (pump) beam, and *P*_2*ω*_ stands for the generated SH beam power, *R* is the repetition rate, *N*_*a*_ = 0.5 is the numerical aperture, *λ*_2_ = 780 nm is the SH wavelength, *τ* = *τ*_2_ = 150 fs are the pulse durations at fundamental and SH wavelengths, $\phi = 8\pi {\int}_0^1 {| {{\rm{co}}{{\rm{s}}^{ - 1}}\rho - \rho {{\sqrt {1 - {\rho ^2}} }|^2}} \rho \,{\rm{d}}\rho = 3.56}$ from ref. ^54^, and ${n_1} = {n_2}\sim 1.45$ are the refractive indexes of the substrate at the wavelengths of the fundamental and SHG, respectively. The effective bulk-like second-order susceptibility of MoS_2_ $( {\chi _{{\rm{eff}}}^{(2)}} )$ can be obtained from Eq. (1) with $\chi _{{\rm{eff}}}^{(2)} = \frac{{\chi _{\rm{s}}^{(2)}}}{{{t_{{\rm{Mo}}{{\rm{S}}_2}}}}}$, where ${t_{{\rm{Mo}}{{\rm{S}}_2}}} = 0.65$ nm is the 1L-MoS_2_ thickness^10, 24^. We obtain the effective second-order susceptibility $\chi _{{\rm{eff}}}^{(2)}\sim 2.2$ pmV^−1^ for 1L-MoS_2_. Reference ^49^ reported a bulk-like second-order susceptibility 29 pmV^−1^, which is ~10 times larger than here. However, several other studies reported ~5 pmV^−1^ for 1560 nm^20, 52, 55^. Thus, our measured $\chi _{{\rm{eff}}}^{(2)}$ agrees well with earlier values measured with similar excitation wavelength.

The third-order susceptibility $\chi _{{\rm{eff}}}^{(3)}$ can be estimated by comparing the measured THG signal from MoS_2_ to that of 1L-graphene (SLG):

2 $$\chi _{{\rm{eff}}}^{(3)} \approx \frac{{{t_{{\rm{SLG}}}}}}{{{t_{{\rm{Mo}}{{\rm{S}}_2}}}}}\sqrt {\frac{{{\rm{TH}}{{\rm{G}}_{{\rm{Mo}}{{\rm{S}}_2}}}}}{{{\rm{TH}}{{\rm{G}}_{{\rm{SLG}}}}}}} \chi _{{\rm{SLG}}}^{(3)} .$$

with *t*_SLG_ = 0.33 nm the SLG thickness, and THG_SLG_ and THG$_{{\rm{Mo}}{{\rm{S}}_{\rm{2}}}}$ the measured signals from SLG and MoS_2_, respectively. Our results show that THG from 1L-MoS_2_ is around three times larger than THG_SLG_, which indicates that *χ* ^(3)^ of 1L-MoS_2_ is comparable to that of SLG, in the frequency range of our experiments. Previous reports indicate^49, 56^ that $\chi_{{\rm{SLG}}} ^{(3)}$ is ~10^−17^–10^−19^ m^2^ V^−2^. Thus, based on Eq. (2), *χ* ^(3)^ of 1L-MoS_2_ is in the same range. This is remarkable, as SLG is known to have a large *χ* ^(3)^ ^56–59^. Reference ^18^ reported *χ* ^(3)^ of 7L-MoS_2_ to be approximately three orders of magnitude smaller than $\chi_{{\rm{SLG}}} ^{(3)}$ of ref. ^56^. $\chi _{{\rm{SLG}}}^{(3)}$ from ref. ^56^ is much higher than other theoretical^58^ and experimental^49^ values. We believe that our measured ratio between 1L-MoS_2_ and SLG is more accurate, since we measured both materials at the same time under the same conditions.

We note that large discrepancies can be found in earlier reported effective susceptibilities for layered materials (LM). For example, there is a approximately four orders of magnitude difference in *χ* ^(3)^ for SLG (~10^−15^ m^2^V^−2^ in ref. ^57^; ~10^−19^ m^2^V^−2^ in ref. ^49^). There is an approximately three orders of magnitude difference in *χ* ^(2)^ reported for 1L-MoS_2_ at 800 nm (e.g., ~10^−7^ mV^−1^ in ref. ^15^; and ~10^−10^ mV^−1^ in ref. ^17^). Effective susceptibilities are well defined only in three-dimensional materials, since their definition involves a polarization per unit volume^1^. Therefore, given the large discrepancies in literature, it is better to describe the nonlinear processes in LMs using the ratio between the harmonic signal power and the incident pump power (i.e., harmonic conversion efficiency). In this case, when comparing the efficiencies in our measurements with those in ref. ^49^, our THG efficiency (~4.76 × 10^−10^) is ~1.4 times larger than that (~3.38 × 10^−10^) in ref. ^49^, while our SHG efficiency (~6.47 × 10^−11^) is twice that of ref. ^49^. Since the effective susceptibilities are not well defined for LMs and also depend on the calculation method, we believe that the conversion efficiency is a better figure of merit for LMs.

## Discussion

Our measurements show that the nonlinear response of 1L-MoS_2_ and SLG are comparable in magnitude, both revealing stronger nonlinear efficiency than three-dimensional nonlinear materials, such as diamond^1^ and quartz^59^. This can be explained by considering their effective Hamiltonians^27, 34–37, 60^. The main contribution to THG is paramagnetic. This is described by the square diagram in Supplementary Notes 1–4 (Supplementary Figs. 1–6). This paramagnetic contribution is mainly related to the strong inter-band coupling in the effective Hamiltonian, controlled by large velocity scales, ${v_{\rm{F}}} \approx \frac{c}{{300}}$ and $v = \frac{{{t_0}{a_0}}}{\hbar } \approx 0.65 \times \frac{c}{{300}}$ for SLG and 1L-MoS_2_, with *c* the speed of light. The SLG paramagnetic third-harmonic efficiency (PTHE) is proportional to the square of third-order conductivity. Since^39^ $\sigma _{yyyy}^{(3)} \propto v_{\rm{F}}^2$, we get an overall prefactor $v_{\rm{F}}^4$, which explains the strong nonlinear SLG response. Similarly, for 1L-MoS_2_, the square diagram contains four paramagnetic current vertices, which gives an overall prefactor *v* ^4^, and an integral over the dummy momentum variables, which gives a prefactor $\frac{1}{{{v^2}}}$ (see Supplementary Note 3). Therefore, the third-order response function, $\Pi _{yyyy}^{(3)}$, is proportional to *v* ^2^, which implies a scaling of PTHE as *v* ^4^. Exciton physics is not considered because our experimental conditions only capture off-resonance transitions.

1L-MoS_2_ is transparent at this wavelength due to its ~1.9 eV gap^12^, while SLG absorbs 2.3% of the light^61^. Therefore, 1L-MoS_2_ and other TMDs are promising for integration with waveguides or fibers for all-optical nonlinear devices, such as all-optical modulators and signal processing devices, where materials with nonlinear properties are essential^11^.

The SHG and THG power dependence follows quadratic and cubic trends, Fig. 4b. At our power levels, THG is up to 30 times stronger than SHG. 1L-TMDs have strongly bound excitons that can modify their optical properties^62–64^. The exciton resonances also affect their nonlinear optical responses^17, 65, 66^. References ^51, 55^ reported that when the SHG energy is above the A and B excitons, resonance effects are not observed. In our experiments, the energy of 3*ω* photons is above the A exciton but does not directly overlap with the A or B excitons. Thus, we do not assign the large THG/SHG intensity ratio to an excitonic enhancement, but to the approximate rotational invariance of the 1L-MoS_2_ band structure at low energies, which is broken by trigonal warping.

SHG is weaker than expected for a non-centrosymmetric material, due to near-isotropic bands contributing to the SHG signal for our low incident photon energies (0.8 eV). Even in the presence of a weak trigonal warping, SHG and THG might be comparable above the threshold for two- and three-photon absorption edges. However, this is not a resonant effect. Resonances only emerge when the laser matches a single level (like an excitonic level) rather than a continuum of states^67^. In our analysis, SHG would be absent without trigonal warping. But, trigonal warping alone cannot explain the magnitude of the FHG signal compared to SHG and THG.

Figure 4c compares the THG/SHG ratio from experiments and calculations based on the ***k***·***p*** theory^35^ (see Supplementary Note 1) and finite-temperature diagrammatic perturbation theory^39^ (see Supplementary Notes 3 and 4). The calculations are a factor 2 smaller than the experiments. Considering the complexity of the nonlinear optical processes and that our calculations ignore high-energy band structure effects^29^ and many-body renormalizations^65^, we believe this to be a satisfactory agreement, indicating the importance of trigonal warping in harmonic generation.

FHG generally derives from cascades of lower-order nonlinear multiphoton processes^68^. With an excitation wavelength of 1560 nm, this could be, e.g., a cascade of two SHG processes, where 780 nm photons are first generated through SHG (*ω*_1560 nm_ + *ω*_1560 nm_ ⇒ *ω*_780 nm_) and then undergo another SHG process (*ω*_780 nm_ + *ω*_780 nm_ ⇒ *ω*_390 nm_). To yield a FHG at 390 nm of the same intensity as SHG at 780 nm in this cascaded process, one would need a conversion efficiency (defined as *P*_2*ω*_/*P*_pump_^1^) for the second SHG process (i.e., *ω*_780 nm_ + *ω*_780 nm_ ⇒ *ω*_390 nm_) to be close to unity. However, we observe a conversion efficiency ~10^−10^ for SHG. Therefore, we conclude that our FHG does not arise from cascaded SHGs. Another possible cascade process is based on THG (*ω*_1560 nm_ + *ω*_1560 nm_ + *ω*_1560 nm_ ⇒ *ω*_520nm_) and sum-frequency generation (*ω*_520 nm_ + *ω*_1560 nm_ ⇒ *ω*_390 nm_). We find that THG strongly increases up to *N* = 5, as for Fig. 4a. Therefore, we expect this cascaded process to have a similar trend with *N*. However, we only observe FHG in 1L-MoS_2_. Thus, we also exclude this cascade process, and conclude that this is a direct *χ* ^(4)^ process.

We now consider the dependence of our results on the elliptical polarization of the incident light. We consider an incident laser beam with arbitrary polarization, i.e., ${\bf{E}} = \left| {\bf{E}} \right|{{\hat {\varepsilon }}_ \pm }$ with ${{{\hat \varepsilon }}_ \pm } = \widehat {\bf{x}}\,{\rm{cos}}(\theta ) \pm i\widehat {\bf{y}}\,{\rm{sin}}(\theta )$. Using the crystal symmetries of 1L-MoS_2_, we derive (see Supplementary Note 2) the following expressions for the second- and third-order polarizations **P** ^(2)^ and **P** ^(3)^:

3 $${{\bf{P}}^{(2)}} = {\epsilon _0}\chi _{yyy}^{(2)}{\left| {\bf{E}} \right|^2}\left[ { \mp i\,{\rm{sin}}(2\theta )\widehat {\bf{x}} - \widehat {\bf{y}}} \right]$$

and

4 $${{\bf{P}}^{(3)}} = {\epsilon _0}\chi _{yyyy}^{(3)}{\left| {\bf{E}} \right|^3}{{{\hat \varepsilon }}_ \pm }{\rm{cos}}(2\theta ) .$$

Note that *θ* = 0° corresponds to a linearly polarized laser along the $\widehat {\bf{x}}$ direction, perpendicular to the $D_{3{\rm{h}}}^1$ mirror symmetry plane, while *θ* = 45° corresponds to a circularly polarized laser. From Eq. (3), we expect the intensity of SHG in response to a circularly polarized pump laser to be twice that of a linearly polarized laser. Equation (4) implies vanishing THG in response to a circularly polarized pump laser.

**Fig. 5:**
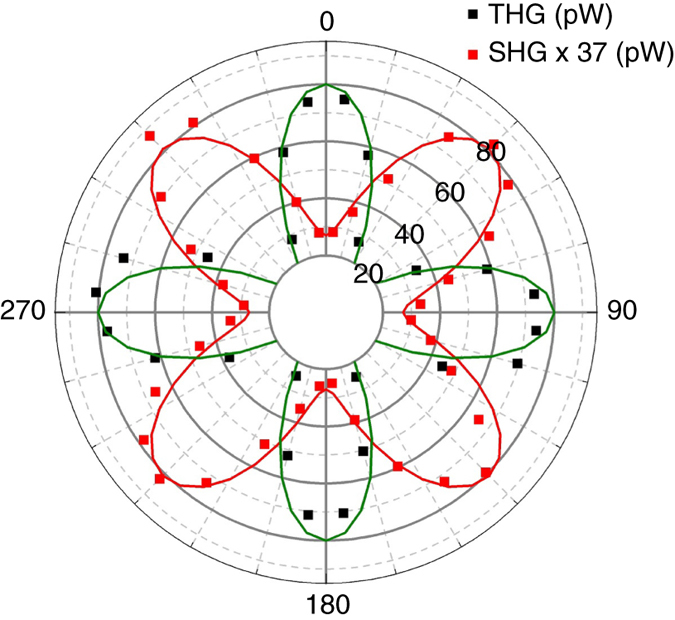
SHG and THG dependence on the pump light polarization. The polar plot angle corresponds to linearly polarized light when *θ* = 0° + *m*·90°, and to circularly polarized pump light when *θ* = 45° + *m*·90°. The SHG power is multiplied by a factor of 37 to fit in the same scale as THG

We measure the dependence of SHG and THG on elliptical polarization using a linearly polarized laser and a rotating QWP. Depending on the angle *θ* between the QWP axes and the laser polarization, the excitation light is linearly (*θ* = 0° + *m*·90°) or circularly (*θ* = 45° + *m*·90°) polarized. Figure 5 shows that the experimental data are in agreement with Eqs. (3) and (4). The THG signal is maximum for a linearly polarized excitation laser, while it vanishes for circularly polarized light. SHG is always visible, but its intensity is maximum for circularly polarized light.

Given that harmonic generation is strongly dependent on the symmetry and stacking of layers and that different 1L-TMDs (e.g., WSe_2_, MoSe_2_) all have similar nonlinear response^11, 14, 15, 21^, one could use heterostructures (e.g., MoS_2_/WSe_2_) to engineer SHG and other nonlinear processes for high photon-conversion efficiency for a wide range of applications requiring the generation of higher frequencies. This may lead to the use of LMs and heterostructures for applications utilizing optical nonlinearities (e.g., all-optical devices, frequency combs, high-order harmonic generation, multiphoton microscopy, and therapy etc.).

## Methods

### Determination of MoS_2_ thickness from SHG and THG signals

**Fig. 6:**
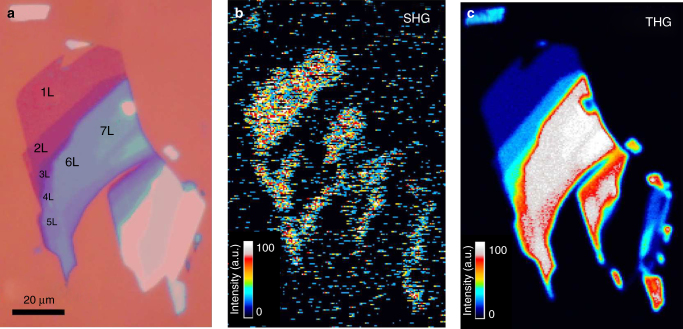
Optical and multiphoton images of few-layer MoS_2_ flake. **a** Optical micrograph, **b** SHG, and **c** THG images of flake with few-layer areas under 1560 nm excitation

SHG and THG for FL-MoS_2_ (*N* = 1…7) are studied on the flakes in Fig. 6a. SHG and THG images are shown in Fig. 6b, c. At 1560 nm, the contrast between 1 and 3L areas is small, as well as the contrast between 3, 5, and 7 L regions (Fig. 6b).

The THG signal increases up to *N* = 7, Figs. 4a and 6c. On the other hand, the SHG signal (Fig. 6b) is only generated in ON flakes, due to symmetry^14^. Therefore, areas with intensity between the 3, 5, and 7L regions in Fig. 6c, but dark in SHG, are 4 and 6 L. The dependence of the intensities of THG and SHG on *N* is plotted in Fig. 4a. The combination of SHG and THG can be used to identify *N* at least up to 7. The THG signal develops as a function of *N*. Using Maxwell’s equations for a nonlinear medium with thickness *t* and considering the slowly varying amplitude approximation^1, 69^, we obtain:

5 $$\frac{{{I_{3\omega }}}}{{{I_{{\rm{in}}}}}} \approx \frac{{{{(3\omega )}^2}I_{{\rm{in}}}^{\rm{2}}}}{{16n_1^3{n_3}\epsilon _0^2{c^4}}}{\left| {{\chi ^{(3)}}( - 3\omega ;\omega ,\omega ,\omega )} \right|^2}{t^2}{\rm{sin}}{{\rm{c}}^2}\left( {\frac{{\Delta kt}}{2}} \right),$$

where *I*_in_ and *I*_3*ω*_ are the intensities of the incident and THG light, respectively, and *χ* ^(3)^(−3*ω*;*ω*, *ω*, *ω*) is the third-order optical susceptibility, ${n_{j = 1,3}} = \sqrt {{\epsilon ^{(1)}}(j \omega )}$, with $\epsilon ^{(1)}$ the TMD linear dielectric function. Δ*kt* is the phase mismatch between the fundamental and third harmonic generated waves.

For Δ*kt* ≈ 0, THG adds up quadratically with light propagation length (i.e., *t* ∝ *N*). The signal starts to saturate for *N* = 6. The possible reasons for subquadratic signal build-up can be either phase mismatch, or absorption^13^. For THG, Δ*k* = 3*k*_in_ ± *k*_3*ω*_, where *k*_in_ and *k*_3*ω*_ are the wavevectors of the incident and THG signals, respectively, where the plus sign indicates THG generated in the backward direction, while minus identifies forward generated THG. Even for backward generated THG, Δ*kt* ≈ 0 for 6L-MoS_2_ (~4.3 nm^70^). This rules out phase mismatch as the origin of the signal saturation when *N* ≤ 6. Therefore, we assume that the signal saturation is due to absorption of the third harmonic light.

### Diagrammatic nonlinear response theory

To quantify theoretically the strength of nonlinear harmonic generation processes, we generalize the diagrammatic perturbation theory approach^39^ to the case of TMDs. We combine this technique with a low-energy ***k***·***p*** model Hamiltonian ${\cal H}\left( {\bf{k}} \right)$ for 1L-MoS_2_^35^. In such low-energy model, light-matter interactions are treated by employing minimal coupling^35, 39^, **k** → **k** + *e* **A**(*t*)/*ħ*, where **A**(*t*) is a time-dependent uniform vector potential. Nonlinear response functions are calculated via the multi-legged Feynman diagrams depicted in Supplementary Figs. 2 and 3.

### Data availability

The data that support the findings of this study are available from the corresponding author on request.

## Electronic supplementary material

Supplementary Information

## References

[1] Boyd, R. W. *Nonlinear Optics* (Academic Press, 2003).

[2] Pavone, F. S. & Campagnola, P. J. *Second Harmonic Generation Imaging* (Taylor & Francis, 2013).

[3] Saleh, B. E. A. & Teich, M. C. *Fundamentals of Photonics* (Academic Press, 2003).

[4] Willner AE, Khaleghi S, Chitgarha MR, Yilmaz OF (2014). All-optical signal processing. J. Lightwave Technol.

[5] Zipfel WR, Williams RM, Webb WW (2003). Nonlinear magic: multiphoton microscopy in the biosciences. Nat. Biotechnol..

[6] Bhawalkar JD, He GS, Prasad PN (1996). Nonlinear multiphoton processes in organic and polymeric materials. Rep. Prog. Phys..

[7] Tsang TYF (1995). Optical third-harmonic generation at interfaces. Phys. Rev. A.

[8] Karvonen L (2015). Investigation of second-and third-harmonic generation in few-layer gallium selenide by multiphoton microscopy. Sci. Rep..

[9] Bonaccorso F, Sun Z, Hasan T, Ferrari AC (2010). Graphene photonics and optoelectronics. Nat. Photon.

[10] Ferrari AC (2015). Science and technology roadmap for graphene, related two-dimensional crystals, and hybrid systems. Nanoscale.

[11] Sun Z, Martinez A, Wang F (2016). Optical modulators with 2D layered materials. Nat. Photon..

[12] Mak KF, Lee C, Hone J, Shan J, Heinz TF (2010). Atomically thin MoS_2_: a new direct-gap semiconductor. Phys. Rev. Lett..

[13] Eda G (2011). Photoluminescence from chemically exfoliated MoS_2_. Nano Lett..

[14] Li Y (2013). Probing symmetry properties of few-layer MoS_2_ and h-BN by optical second-harmonic generation. Nano Lett..

[15] Kumar N (2013). Second harmonic microscopy of monolayer MoS_2_. Phys. Rev. B.

[16] Wang K (2013). Ultrafast saturable absorption of two-dimensional MoS_2_ nanosheets. ACS Nano.

[17] Malard LM, Alencar TV, Barboza APM, Mak KF, de Paula AM (2013). Observation of intense second harmonic generation from MoS_2_ atomic crystals. Phys. Rev. B.

[18] Wang R (2013). Third-harmonic generation in ultrathin films of MoS_2_. ACS Appl. Mater. Interfaces.

[19] Trolle ML, Seifert G, Pedersen TG (2014). Theory of excitonic second-harmonic generation in monolayer MoS_2_. Phys. Rev. B.

[20] Clark D (2014). Strong optical nonlinearity of CVD-grown MoS_2_ monolayer as probed by wavelength-dependent second-harmonic generation. Phys. Rev. B.

[21] Seyler KL (2015). Electrical control of second-harmonic generation in a WSe_2_ monolayer transistor. Nat. Nanotechnol.

[22] Kuc A, Zibouche N, Heine T (2011). Influence of quantum confinement on the electronic structure of the transition metal sulfide TS_2_. Phys. Rev. B.

[23] Kadantsev ES, Hawrylak P (2012). Electronic structure of a single MoS_2_ monolayer. Solid State Commun.

[24] Wang QH, Kalantar-Zadeh K, Kis A, Coleman JN, Strano MS (2012). Electronics and optoelectronics of two-dimensional transition metal dichalcogenides. Nat. Nanotechnol.

[25] Shi H, Pan H, Zhang Y-W, Yakobson BI (2013). Quasiparticle band structures and optical properties of strained monolayer MoS_2_ and WS_2_. Phys. Rev. B.

[26] Zahid F, Liu L, Zhu Y, Wang J, Guo H (2013). A generic tight-binding model for monolayer, bilayer and bulk MoS_2_. AIP Adv..

[27] Kormányos A (2013). Monolayer MoS_2_: trigonal warping, the *γ* valley, and spin-orbit coupling effects. Phys. Rev. B.

[28] Qiu DY, Felipe H, Louie SG (2013). Optical spectrum of MoS_2_: many-body effects and diversity of exciton states. Phys. Rev. Lett..

[29] Gibertini M, Pellegrino FM, Marzari N, Polini M (2014). Spin-resolved optical conductivity of two-dimensional group-VIb transition-metal dichalcogenides. Phys. Rev. B.

[30] Margulis VA, Muryumin EE, Gaiduk EA (2013). Optical second-harmonic generation from two-dimensional hexagonal crystals with broken space inversion symmetry. J. Phys. Condens. Matter.

[31] Wu S (2012). Quantum-enhanced tunable second-order optical nonlinearity in bilayer graphene. Nano Lett..

[32] Brun SJ, Pedersen TG (2015). Intense and tunable second-harmonic generation in biased bilayer graphene. Phys. Rev. B.

[33] Hipolito F, Pedersen TG, Pereira VM (2016). Nonlinear photocurrents in two-dimensional systems based on graphene and boron nitride. Phys. Rev. B.

[34] Xiao D, Liu G-B, Feng W, Xu X, Yao W (2012). Coupled spin and valley physics in monolayers of MoX_2_ and other group-vi dichalcogenides. Phys. Rev. Lett..

[35] Rostami H, Roldán R, Cappelluti E, Asgari R, Guinea F (2015). Theory of strain in single-layer transition metal dichalcogenides. Phys. Rev. B.

[36] Rostami H, Asgari R, Guinea F (2016). Edge modes in zigzag and armchair ribbons of monolayer MoS_2_. J. Phys. Condens. Matter.

[37] Rostami H, Moghaddam AG, Asgari R (2013). Effective lattice hamiltonian for monolayer MoS_2_: tailoring electronic structure with perpendicular electric and magnetic fields. Phys. Rev. B.

[38] Alidoust N (2014). Observation of monolayer valence band spin-orbit effect and induced quantum well states in MoX_2_. Nat. Commun.

[39] Rostami H, Polini M (2016). Theory of third-harmonic generation in graphene: a diagrammatic approach. Phys. Rev. B.

[40] Bonaccorso F (2012). Production and processing of graphene and 2d crystals. Mater. Today.

[41] Sundaram, R. et al. Electroluminescence in single layer MoS_2_. *Nano Lett*. **13**, 1416–1421 (2013). 10.1021/nl400516a 23514373

[42] Casiraghi C (2007). Rayleigh imaging of graphene and graphene layers. Nano Lett..

[43] Zhang X (2013). Raman spectroscopy of shear and layer breathing modes in multilayer MoS_2_. Phys. Rev. B.

[44] Säynätjoki A (2013). Rapid large-area multiphoton microscopy for characterization of graphene. ACS Nano.

[45] Kieu K, Jones RJ, Peyghambarian N (2010). Generation of few-cycle pulses from an amplified carbon nanotube mode-locked fiber laser system. IEEE Photon. Technol. Lett..

[46] Kieu K, Jones RJ, Peyghambarian N (2010). High power femtosecond source near 1 micron based on an all-fiber er-doped mode-locked laser. Opt. Express.

[47] Miller RC (1964). Optical second harmonic generation in piezoelectric crystals. Appl. Phys. Lett..

[48] Chin AH, Calderón OG, Kono J (2001). Extreme midinfrared nonlinear optics in semiconductors. Phys. Rev. Lett..

[49] Woodward RI (2017). Characterization of the second- and third-order nonlinear optical susceptibilities of monolayer MoS_2_ using multiphoton microscopy. 2D Mater.

[50] Säynätjoki, A. et al. Ultra-strong nonlinear optical processes and trigonal warping in MoS_2_ layers. Preprint at https://arxiv.org/abs/1608.04101 (2016). 10.1038/s41467-017-00749-4 PMC5715017 29026087

[51] Clark D (2015). Near bandgap second-order nonlinear optical characteristics of MoS_2_ monolayer transferred on transparent substrates. Appl. Phys. Lett..

[52] Le CT (2017). Impact of selenium doping on resonant second harmonic generation in monolayer MoS_2_. ACS Photon..

[53] Liu H (2017). High-harmonic generation from an atomically thin semiconductor. Nat. Phys.

[54] Janisch, C. et al. Extraordinary second harmonic generation in tungsten disulfide monolayers. *Sci. Rep*. **4**, 5530 (2014). 10.1038/srep05530 PMC4078302 24984953

[55] Le CT (2016). Nonlinear optical characteristics of monolayer MoSe_2_. Ann. Phys.

[56] Kumar N (2013). Third harmonic generation in graphene and few-layer graphite films. Phys. Rev. B.

[57] Hendry E, Hale PJ, Moger J, Savchenko A, Mikhailov S (2010). Coherent nonlinear optical response of graphene. Phys. Rev. Lett..

[58] Cheng J, Vermeulen N, Sipe J (2014). Third order optical nonlinearity of graphene. New J. Phys..

[59] Hong S-Y (2013). Optical third-harmonic generation in graphene. Phys. Rev. X.

[60] Neto AHC, Guinea F, Peres NM, Novoselov KS, Geim AK (2009). The electronic properties of graphene. Rev. Mod. Phys..

[61] Nair RR (2008). Fine structure constant defines visual transparency of graphene. Science.

[62] Ramasubramaniam A (2012). Large excitonic effects in monolayers of molybdenum and tungsten dichalcogenides. Phys. Rev. B.

[63] Cheiwchanchamnangij T, Lambrecht WRL (2012). Quasiparticle band structure calculation of monolayer, bilayer, and bulk MoS_2_. Phys. Rev. B.

[64] Mak KF (2013). Tightly bound trions in monolayer MoS_2_. Nat. Mater..

[65] Grüning M, Attaccalite C (2014). Second harmonic generation in h-BN and MoS_2_ monolayers: role of electron-hole interaction. Phys. Rev. B.

[66] Wang G (2015). Giant enhancement of the optical second-harmonic emission of WSe_2_ monolayers by laser excitation at exciton resonances. Phys. Rev. Lett..

[67] Haug, H. & Koch, S. W. *Quantum Theory of the Optical and Electronic Properties of Semiconductors* (World Scientific Publishing, 2009).

[68] Zhu S-n, Zhu Y-y, Ming N-b (1997). Quasi-phase-matched third-harmonic generation in a quasi-periodic optical superlattice. Science.

[69] Butcher, P. N. & Cotter, D. *The Elements of Nonlinear Optics* (Cambrige University Press, 1990).

[70] Radisavljevic B, Radenovic A, Brivio J, Giacometti IV, Kis A (2011). Single-layer MoS_2_ transistors. Nat. Nanotechnol.

