# Supplementary material for: Ultra-strong nonlinear optical processes and trigonal warping in MoS2 layers
Source: Nat Commun. 2017 Oct 12;8:893. doi: 10.1038/s41467-017-00749-4 (PMC5715017; doi:10.1038/s41467-017-00749-4)
Supplement: Supplementary file 1 — Supplementary Information [file 41467_2017_749_MOESM1_ESM.pdf]

Supplementary Note 1 Continuum-model Hamiltonian and current matrix elements for 1L-MoS$_2$

For 1L-MoS$_2$ we use the low-energy $\boldsymbol{k}\cdot\boldsymbol{p}$ continuum-model Hamiltonian described in Ref.[1]. Around the K and K$'$ points the model Hamiltonian contains isotropic $\mathcal{H}_{\rm i}$ and trigonal warping $\mathcal{H}_{\rm tw}$ contributions, i.e. $\mathcal{H}=\mathcal{H}_{\rm i}+\mathcal{H}_{\rm tw}$, with:

$$\mathcal{H}_{\rm i}(\mathbf{k},\tau,s)=\frac{\lambda_0\tau s}{2}+\frac{\Delta+\lambda\tau s}{2}\sigma_z+t_0a_0\mathbf{k}\cdot\boldsymbol{\sigma}_\tau+\frac{\hbar^2|\mathbf{k}|^2}{4m_0}(\alpha+\beta\sigma_z)\ , \tag{1}$$

and

$$\mathcal{H}_{\rm tw}(\mathbf{k},\tau,s)=t_1a_0^2(\mathbf{k}\cdot\boldsymbol{\sigma}_\tau^*)\sigma_x(\mathbf{k}\cdot\boldsymbol{\sigma}_\tau^*)+t_2a_0^3\tau(k_x^3-3k_xk_y^2)(\alpha'+\beta'\sigma_z)\ . \tag{2}$$

Here, $s=\pm$ is a spin index, $\tau=\pm$ is a valley index, and $\boldsymbol{\sigma}_\tau=(\tau\sigma_x,\sigma_y)$, with σ_x and σ_y ordinary 2×2 Pauli matrices operating on a suitable conduction/valence band basis[1]. We note that the terms in the Hamiltonian that contain the parameters Δ, β, β' and λ_0 are related to broken spatial inversion symmetry in 1L-MoS$_2$. The trigonal warping term contains three parameters, α',β', and t_1. The contribution to the band dispersion due to trigonal warping has the characteristic form $z_\pm\cos(3\phi)$, where $z_\pm=t_2(\alpha'\pm\beta')\pm 4t_0t_1/\left[2\Delta-(\lambda_0-\lambda)\tau s\right]$, and z_+ (z_-) stands for conduction (valence) band[2]. According to *ab-initio* calculations[3, 4], symmetry considerations[4, 5], and experimental evidence[6], the valence band of 1L-MoS$_2$ is strongly warped, while the conduction band is nearly isotropic.

The Hamiltonian $\mathcal{H}$ can be diagonalized. Eigenvalues $\epsilon^{\rm c(v)}_{\mathbf{k},\tau,s}$ and eigenvectors $|u^{\rm c(v)}_{\mathbf{k},\tau,s}\rangle$ are:

$$\epsilon^{\rm c(v)}_{\mathbf{k},\tau,s}=h_0(\mathbf{k},\tau,s)\pm\sqrt{\left[h_z(\mathbf{k},\tau,s)\right]^2+|h_{12}(\mathbf{k},\tau,s)|^2} \tag{3}$$

and

$$|u^{\rm c(v)}_{\mathbf{k},\tau,s}\rangle=\frac{1}{\sqrt{\left[D^{\rm c(v)}(\mathbf{k},\tau,s)\right]^2+|h_{12}(\mathbf{k},\tau,s)|^2}}\begin{bmatrix}-h_{12}(\mathbf{k},\tau,s)\\ D^{\rm c(v)}(\mathbf{k},\tau,s)\end{bmatrix}\ , \tag{4}$$

where

$$h_0(\mathbf{k},\tau,s)=\frac{\lambda_0}{2}\tau s+\frac{\hbar^2k^2}{4m_0}\alpha+t_2a_0^3\tau(k_x^3-3k_xk_y^2)\alpha'\ , \tag{5}$$

$$h_z(\mathbf{k},\tau,s)=\frac{\Delta+\lambda\tau s}{2}+\frac{\hbar^2k^2}{4m_0}\beta+t_2a_0^3\tau(k_x^3-3k_xk_y^2)\beta'\ , \tag{6}$$

$$h_{12}(\mathbf{k},\tau,s)=t_0a_0(\tau k_x-ik_y)+t_1a_0^2(\tau k_x+ik_y)^2\ , \tag{7}$$

and

$$D^{\rm c(v)}(\mathbf{k},\tau,s) = h_z(\mathbf{k},\tau,s) \mp \sqrt{[h_z(\mathbf{k},\tau,s)]^2 + |h_{12}(\mathbf{k},\tau,s)|^2}~. \tag{8}$$

We need the matrix elements of the current operator for the evaluation of the nonlinear response functions. We start by introducing the so-called paramagnetic current operator[7] ($c = 1$, where c is the speed of light, $-e < 0$ is the electron charge):

$$j_\ell(\mathbf{k}) \equiv -\left.\frac{\delta\mathcal{H}(\mathbf{k}+e\mathbf{A}/\hbar)}{\delta A_\ell}\right|_{\mathbf{A}=\mathbf{0}} = -\frac{e}{\hbar}\frac{\partial\mathcal{H}}{\partial k_\ell}~, \tag{9}$$

where $\ell = x, y$ is a Cartesian index. The diamagnetic contributions to the current operator can be written as follows:

$$\kappa_{\ell_1\ell_2}(\mathbf{k}) \equiv -\left.\frac{\delta^2\mathcal{H}(\mathbf{k}+e\mathbf{A}/\hbar)}{\delta A_{\ell_1}\delta A_{\ell_2}}\right|_{\mathbf{A}=\mathbf{0}} = -\left(\frac{e}{\hbar}\right)^2\frac{\partial^2\mathcal{H}}{\partial k_{\ell_1}\partial k_{\ell_2}} \tag{10}$$

and

$$\xi_{\ell_1\ell_2\ell_3}(\mathbf{k}) \equiv -\left.\frac{\delta^3\mathcal{H}(\mathbf{k}+e\mathbf{A}/\hbar)}{\delta A_{\ell_1}\delta A_{\ell_2}\delta A_{\ell_3}}\right|_{\mathbf{A}=\mathbf{0}} = -\left(\frac{e}{\hbar}\right)^3\frac{\partial^3\mathcal{H}}{\partial k_{\ell_1}\partial k_{\ell_2}\partial k_{\ell_3}} \tag{11}$$

Using the continuum-model Hamiltonian introduced in Supplementary Equations (1) and (2), we find:

$$j_\ell = -\frac{e}{\hbar}\left\{\frac{\partial h_0}{\partial k_\ell} + \frac{\partial h_z}{\partial k_\ell}\sigma_z + {\rm Re}[\frac{\partial h_{12}}{\partial k_\ell}]\sigma_x - {\rm Im}[\frac{\partial h_{12}}{\partial k_\ell}]\sigma_y\right\} \tag{12}$$

and

$$\kappa_{\ell\ell} = -\left(\frac{e}{\hbar}\right)^2\left\{\frac{\partial^2 h_0}{\partial k_\ell^2} + \frac{\partial^2 h_z}{\partial k_\ell^2}\sigma_z + {\rm Re}[\frac{\partial^2 h_{12}}{\partial k_\ell^2}]\sigma_x - {\rm Im}[\frac{\partial^2 h_{12}}{\partial k_\ell^2}]\sigma_y\right\}~. \tag{13}$$

Similarly, one can derive an explicit expression for $\xi_{\ell\ell\ell}$.

The required matrix elements of j_ℓ and $\kappa_{\ell\ell}$ between the eigenspinors given in Supplementary Equation (4) read:

$$\begin{aligned} j_\ell^{\rm cv}(\mathbf{k},\tau,s) &\equiv \langle u^{\rm c}_{\mathbf{k},\tau,s}|j_\ell|u^{\rm v}_{\mathbf{k},\tau,s}\rangle \\ &= \frac{e}{\hbar}\left\{\frac{h_z(\mathbf{k},\tau,s){\rm Re}\left[h_{12}(\mathbf{k},\tau,s)\partial h^*_{12}(\mathbf{k},\tau,s)/\partial k_\ell\right]}{|h_{12}(\mathbf{k},\tau,s)|\sqrt{[h_z(\mathbf{k},\tau,s)]^2+|h_{12}(\mathbf{k},\tau,s)|^2}}\right. \\ &+ i\frac{{\rm Im}\left[h_{12}(\mathbf{k},\tau,s)\partial h^*_{12}(\mathbf{k},\tau,s)/\partial k_\ell\right]}{|h_{12}(\mathbf{k},\tau,s)|} \\ &- \left.\frac{|h_{12}(\mathbf{k},\tau,s)|\partial h_z(\mathbf{k},\tau,s)/\partial k_\ell}{\sqrt{[h_z(\mathbf{k},\tau,s)]^2+|h_{12}(\mathbf{k},\tau,s)|^2}}\right\}~, \end{aligned} \tag{14}$$

$$j_\ell^{\mathrm{cc(vv)}}(\mathbf{k},\tau,s) \equiv \langle u^{\mathrm{c(v)}}_{\mathbf{k},\tau,s}|j_\ell|u^{\mathrm{c(v)}}_{\mathbf{k},\tau,s}\rangle = -\frac{e}{\hbar}\left\{\frac{\partial h_0(\mathbf{k},\tau,s)}{\partial k_\ell}\right.$$

$$\left.\pm \frac{h_z(\mathbf{k},\tau,s)\partial h_z(\mathbf{k},\tau,s)/\partial k_\ell + \mathrm{Re}\left[h_{12}(\mathbf{k},\tau,s)\partial h^*_{12}(\mathbf{k},\tau,s)/\partial k_\ell\right]}{\sqrt{[h_z(\mathbf{k},\tau,s)]^2+|h_{12}(\mathbf{k},\tau,s)|^2}}\right\}, \tag{15}$$

$$\begin{aligned}\kappa^{\mathrm{cv}}_{\ell\ell}(\mathbf{k},\tau,s) &\equiv \langle u^{\mathrm{c}}_{\mathbf{k},\tau,s}|\kappa_{\ell\ell}|u^{\mathrm{v}}_{\mathbf{k},\tau,s}\rangle \\ &= \left(\frac{e}{\hbar}\right)^2\left\{\frac{h_z(\mathbf{k},\tau,s)\mathrm{Re}\left[h_{12}(\mathbf{k},\tau,s)\partial^2 h^*_{12}(\mathbf{k},\tau,s)/\partial k_\ell^2\right]}{|h_{12}(\mathbf{k},\tau,s)|\sqrt{[h_z(\mathbf{k},\tau,s)]^2+|h_{12}(\mathbf{k},\tau,s)|^2}}\right. \\ &+ i\frac{\mathrm{Im}\left[h_{12}(\mathbf{k},\tau,s)\partial^2 h^*_{12}(\mathbf{k},\tau,s)/\partial k_\ell^2\right]}{|h_{12}(\mathbf{k},\tau,s)|} \\ &- \left.\frac{|h_{12}(\mathbf{k},\tau,s)|\partial^2 h_z(\mathbf{k},\tau,s)/\partial k_\ell^2}{\sqrt{[h_z(\mathbf{k},\tau,s)]^2+|h_{12}(\mathbf{k},\tau,s)|^2}}\right\},\end{aligned} \tag{16}$$

and

$$\kappa_{\ell\ell}^{\mathrm{cc(vv)}}(\mathbf{k},\tau,s) \equiv \langle u^{\mathrm{c(v)}}_{\mathbf{k},\tau,s}|\kappa_{\ell\ell}|u^{\mathrm{c(v)}}_{\mathbf{k},\tau,s}\rangle = -\left(\frac{e}{\hbar}\right)^2\left\{\frac{\partial^2 h_0(\mathbf{k},\tau,s)}{\partial k_\ell^2}\right.$$

$$\left.\pm \frac{h_z(\mathbf{k},\tau,s)\partial^2 h_z(\mathbf{k},\tau,s)/\partial k_\ell^2 + \mathrm{Re}\left[h_{12}(\mathbf{k},\tau,s)\partial^2 h^*_{12}(\mathbf{k},\tau,s)/\partial k_\ell^2\right]}{\sqrt{[h_z(\mathbf{k},\tau,s)]^2+|h_{12}(\mathbf{k},\tau,s)|^2}}\right\}. \tag{17}$$

We note that intra-band matrix elements (e.g. j_y^{cc} and $\kappa_{yy}^{\mathrm{cc}}$) have a definite parity while inter-band ones (e.g. j_y^{cv} and $\kappa_{yy}^{\mathrm{cv}}$) do not respect the parity symmetry. This fact is at the origin of the vanishing of the paramagnetic contribution to even harmonic-generation response functions. Therefore, as we will see later, only diamagnetic terms yield a finite contribution to even harmonic-generation responses.

Supplementary Note 2 General symmetry considerations

Our continuum-model Hamiltonian is derived from a tight-binding Hamiltonian in which the zigzag direction of the lattice coincides with the $\hat{\mathbf{x}}$ direction. The zigzag direction is perpendicular to the reflection (mirror) symmetry plane of the 1L-MoS_2 lattice (see Supplementary Figure 1).

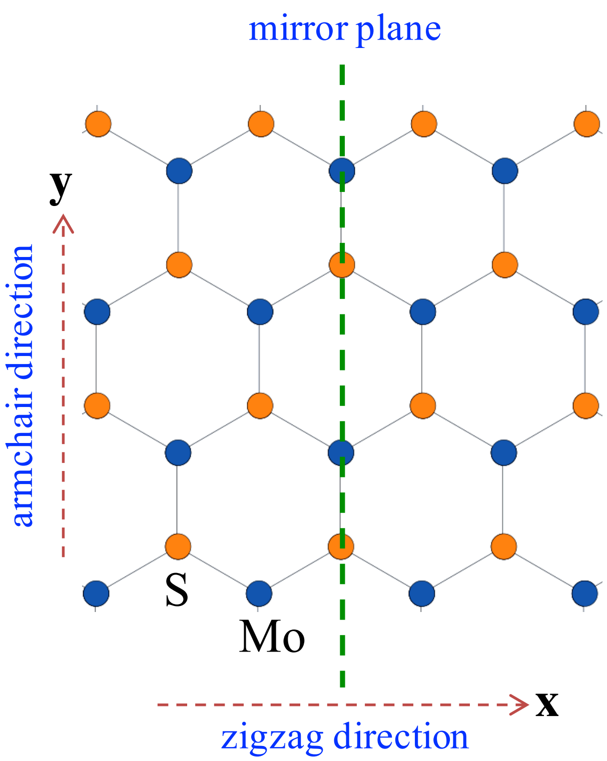

Supplementary Figure 1: Top view of the 1L-MoS_2 lattice.

The n-th order optical susceptibilities $\chi^{(n)}_{\ell i_1 i_2 \dots i_n}$ are defined as:

$$P^{(n)}_{\ell}(\omega_\Sigma) = \epsilon_0 \sum_{i_1 i_2 \dots i_n} \chi^{(n)}_{\ell i_1 i_2 \dots i_n}(-\omega_\Sigma; \omega_1, \omega_2, \dots, \omega_n) E_{i_1}(\omega_1) E_{i_2}(\omega_2) \dots E_{i_n}(\omega_n) \ , \tag{18}$$

where E_i and $P^{(n)}_\ell$ are the Cartesian components of the electric field $\mathbf{E}$ and the n-th order macroscopic polarization $\mathbf{P}^{(n)}$, respectively, and ϵ_0 is the vacuum permittivity. Note that i_1, i_2, $\dots$, i_n are Cartesian indices and $\omega_\Sigma \equiv \sum_i \omega_i$.

Since 1L-MoS_2 belongs to the $D^1_{3\mathrm{h}}$ symmetry group, the only non-vanishing elements of the second-order susceptibility are[8]:

$$\chi^{(2)}_{yyy} = -\chi^{(2)}_{yxx} = -\chi^{(2)}_{xxy} = -\chi^{(2)}_{xyx} \ , \tag{19}$$

while for the case of the third-order response we have[8]:

$$\chi^{(3)}_{yyyy} = \chi^{(3)}_{xxxx} = \chi^{(3)}_{yyxx} + \chi^{(3)}_{yxxy} + \chi^{(3)}_{yxyx} \ , \tag{20}$$

and

$$\begin{aligned}
\chi^{(3)}_{xxyy} &= \chi^{(3)}_{yyxx} \ , \\
\chi^{(3)}_{xyyx} &= \chi^{(3)}_{yxxy} \ , \\
\chi^{(3)}_{xyxy} &= \chi^{(3)}_{yxyx} \ .
\end{aligned} \tag{21}$$

In the case of a linearly-polarized pump laser, we expect a SHG maximum when the laser is polarized along the $\hat{\mathbf{y}}$ direction, i.e. perpendicular to the zigzag direction. On the contrary, if the incident light is polarized along the $\hat{\mathbf{x}}$ direction, i.e. the zigzag direction, we expect a vanishing SHG signal due to the reflection symmetry (i.e. $\sigma_{\rm v} : x \to -x$) along this axis. Our continuum-model Hamiltonian is consistent with these general expectations based on symmetry and we therefore find $\chi^{(2)}_{xxx} = 0$, even in the presence of trigonal warping. This is because the contribution in the two valleys identically cancel each other.

Using Supplementary Equations (18),(19),(20) and (21) we obtain Eqs. (3),(4) of the main text, which describe the dependence between induced charge polarization, $\mathbf{P}$, and the polarization of the incident laser. In the case of a circularly-polarized pump laser, we have $\mathbf{E} = |\mathbf{E}|\hat{\varepsilon}_{\pm}$ with $\hat{\boldsymbol{\varepsilon}}_{\pm} = (\hat{\mathbf{x}} \pm i\hat{\mathbf{y}})/\sqrt{2}$. Using Eqs. (3),(4) of the main text we arrive at the following results for the circularly-polarized pump laser:

$$\mathbf{P}^{(2)} = \mp i\sqrt{2}\epsilon_0 \chi^{(2)}_{yyy} |\boldsymbol{E}|^2 \hat{\boldsymbol{\varepsilon}}_{\mp} \tag{22}$$

and

$$\mathbf{P}^{(3)} = 0 \ . \tag{23}$$

Supplementary Equation (22) implies an *opposite* polarization of the SHG signal with respect to the pump laser, while Supplementary Equation (23) implies no THG signal in response to a circularly-polarized pump laser.

For quantitative results only the following three tensor elements: $\chi^{(2)}_{yyy}$, $\chi^{(3)}_{yyyy}$ and $\chi^{(4)}_{yyyyy}$ are required for second-, third- and fourth-order nonlinear response functions, respectively.

Supplementary Note 3 Nonlinear response functions

The response of an electron system to light can be calculated by adopting different gauges for describing the electric field of incident light. The gauge in which a uniform electric field $\mathbf{E}(t)$ is described in terms of a uniform time-dependent vector potential, $\mathbf{E}(t) = -\partial \mathbf{A}(t)/\partial t$, is convenient in solids as it does not break Bloch translational invariance. The vector potential

couples to matter degrees of freedom through the minimal coupling, i.e. $\mathbf{k} \to \mathbf{k} + e\mathbf{A}/\hbar$. The external vector potential induces a current $\mathbf{J}(t)$, which can be expanded in a power series of $\mathbf{A}(t)$. For each Cartesian component, we write $J_\ell = \sum_n J_\ell^{(n)}$ where n denotes the n-th order in powers of $\mathbf{A}(t)$. In Fourier transform with respect to time we therefore obtain:

$$J_\ell^{(n)}(\omega_\Sigma) \equiv \sum_{i_1,i_2,\ldots,i_n} \Pi^{(n)}_{\ell i_1 i_2 \ldots i_n}(-\omega_\Sigma;\omega_1,\omega_2,\ldots,\omega_n) A_{i_1}(\omega_1) A_{i_2}(\omega_2) \ldots A_{i_n}(\omega_n) \ , \tag{24}$$

where $\mathbf{A}(\omega_i) = -i\mathbf{E}(\omega_i)/(\omega_i + i\eta/\hbar)$ and η is an infinitesimal positive real number, which is needed to make sure that the external field is absent in the remote past ($t \to -\infty$).

Since the macroscopic current is related to the macroscopic polarization by $\mathbf{J}(t) = \partial \mathbf{P}/\partial t$ [9], we get $\mathbf{J}^{(n)}(\omega_\Sigma) = -i(\omega_\Sigma + i\eta/\hbar)\mathbf{P}^{(n)}(\omega_\Sigma)$, for each order in perturbation theory.

We finally find the following relation between nonlinear response functions and optical susceptibilities:

$$\epsilon_0 \chi^{(n)}_{\ell i_1 i_2 \ldots i_n}(-\omega_\Sigma;\omega_1,\ldots,\omega_n) = i(-i)^n \frac{\Pi^{(n)}_{\ell i_1 i_2 \ldots i_n}(-\omega_\Sigma;\omega_1,\ldots,\omega_n)}{(\omega_\Sigma + i\eta/\hbar)(\omega_n + i\eta/\hbar)\ldots(\omega_1 + i\eta/\hbar)} \ . \tag{25}$$

The n-th order nonlinear response function $\Pi^{(n)}_{\ell i_1 i_2 \ldots i_n}$ contains both paramagnetic and diamagnetic current contributions, which will be denoted by $\Pi^{(n),\mathrm{P}}_{\ell i_1 i_2 \ldots i_n}$ and $\Pi^{(n),\mathrm{D}}_{\ell i_1 i_2 \ldots i_n}$, respectively. The paramagnetic current correlators, which are diagrammatically illustrated in Supplementary Figure 2, read:

$$\Pi^{(1),\mathrm{P}}_{\ell i_1}(i\nu) \equiv \left\langle \hat{j}_{i_1}(-i\nu)\hat{j}_\ell(i\nu) \right\rangle \ , \tag{26}$$

$$\Pi^{(2),\mathrm{P}}_{\ell i_1 i_2}(-i\nu_\Sigma; i\nu_1, i\nu_2) \equiv {\sum_{\mathcal{P}}}' \left\langle \hat{j}_{i_1}(-i\nu_1)\hat{j}_{i_2}(-i\nu_2)\hat{j}_\ell(i\nu_\Sigma) \right\rangle \ , \tag{27}$$

$$\Pi^{(3),\mathrm{P}}_{\ell i_1 i_2 i_3}(-i\nu_\Sigma; i\nu_1, i\nu_2, i\nu_3) \equiv {\sum_{\mathcal{P}}}' \left\langle \hat{j}_{i_1}(-i\nu_1)\hat{j}_{i_2}(-i\nu_2)\hat{j}_{i_3}(-i\nu_3)\hat{j}_\ell(i\nu_\Sigma) \right\rangle \ , \tag{28}$$

and

$$\begin{aligned} &\Pi^{(4),\mathrm{P}}_{\ell i_1 i_2 i_3 i_4}(-i\nu_\Sigma; i\nu_1, i\nu_2, i\nu_3, i\nu_4) \\ \equiv &{\sum_{\mathcal{P}}}' \left\langle \hat{j}_{i_1}(-i\nu_1)\hat{j}_{i_2}(-i\nu_2)\hat{j}_{i_3}(-i\nu_3)\hat{j}_{i_4}(-i\nu_4)\hat{j}_\ell(i\nu_\Sigma) \right\rangle \ . \end{aligned} \tag{29}$$

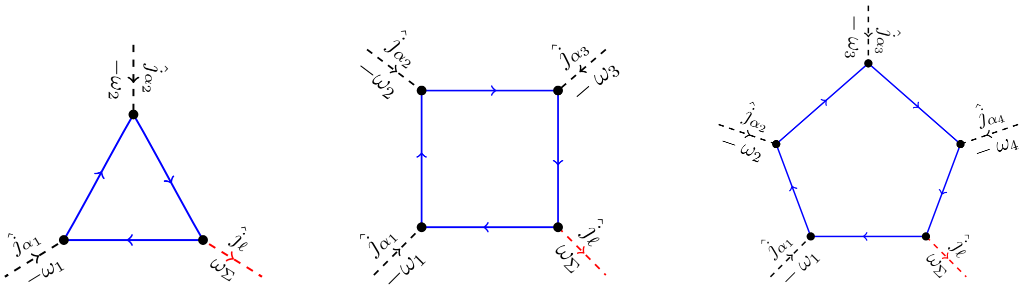

Supplementary Figure 2: Three-, four-, and five-leg Feynman diagrams for the second-, third-, and fourth-order nonlinear *paramagnetic* response functions. Solid lines denote electron propagators while dashed lines denote photons. The quantities $\omega_1 = \cdots = \omega_4 = \omega$ indicate the incoming photon frequencies, while $\hat{j}_\alpha$ denotes the α-th Cartesian component of the paramagnetic current operator.

Here, $\langle \dots \rangle$ denotes the thermal averaging[7, 10], $\hat{j}_i$ indicates the second-quantized form of i-th Cartesian component of the paramagnetic current operator, $\sum'_{\mathcal{P}}$ enforces the so-called "*intrinsic permutation symmetry*" among all dummy variables (i_n, ν_n)[11], and $\nu_\Sigma = \sum_i \nu_i$, where $\nu_i = 2\pi n/\beta$'s are bosonic Matsubara energies corresponding to the incident photon energies. Here, n is a relative integer and $\beta = 1/(k_{\rm B}T)$, with T the electron temperature.

The paramagnetic current correlators in Supplementary Equations (26)-(29) can be calculated by using many-body diagrammatic perturbation theory[12, 13]. Following Ref.[13], we first perform the summation over the fermionic Matsubara energies and then carry out the analytical continuation $\nu_i = \nu \to \hbar\omega + i\eta$ where $\eta \to 0^+$. We find the following relations for the case of $\ell = i_n = y$:

$$\Pi^{(1),{\rm P}}_{yy}(\omega) = \sum_{{\bf k},\tau,s} \sum_{\{\lambda_i\}} U_{\lambda_1\lambda_2} j_y^{\lambda_2\lambda_1} j_y^{\lambda_1\lambda_2} \ , \tag{30}$$

$$\Pi^{(2),{\rm P}}_{yyy}(-2\omega;\omega,\omega) = \sum_{{\bf k},\tau,s} \sum_{\{\lambda_i\}} \frac{j_y^{\lambda_3\lambda_2} j_y^{\lambda_2\lambda_1} j_y^{\lambda_1\lambda_3}}{2(\hbar\omega + i\eta) + \epsilon^{\lambda_1}_{{\bf k},\tau,s} - \epsilon^{\lambda_3}_{{\bf k},\tau,s}} \left(U_{\lambda_1\lambda_2} - U_{\lambda_2\lambda_3}\right) \ , \tag{31}$$

$$\Pi^{(3),\mathrm{P}}_{yyyy}(-3\omega;\omega,\omega,\omega) = \sum_{\mathbf{k},\tau,s}\sum_{\{\lambda_i\}} \frac{j_y^{\lambda_4\lambda_3} j_y^{\lambda_3\lambda_2} j_y^{\lambda_2\lambda_1} j_y^{\lambda_1\lambda_4}}{3(\hbar\omega + i\eta) + \epsilon^{\lambda_1}_{\mathbf{k},\tau,s} - \epsilon^{\lambda_4}_{\mathbf{k},\tau,s}} \times$$
$$\left\{ \frac{U_{\lambda_1\lambda_2} - U_{\lambda_2\lambda_3}}{2(\hbar\omega + i\eta) + \epsilon^{\lambda_1}_{\mathbf{k},\tau,s} - \epsilon^{\lambda_3}_{\mathbf{k},\tau,s}} - \frac{U_{\lambda_2\lambda_3} - U_{\lambda_3\lambda_4}}{2(\hbar\omega + i\eta) + \epsilon^{\lambda_2}_{\mathbf{k},\tau,s} - \epsilon^{\lambda_4}_{\mathbf{k},\tau,s}} \right\} , \tag{32}$$

and

$$\Pi^{(4),\mathrm{P}}_{yyyyy}(-4\omega;\omega,\omega,\omega,\omega) = \sum_{\mathbf{k},\tau,s}\sum_{\{\lambda_i\}} \frac{j_y^{\lambda_5\lambda_4} j_y^{\lambda_4\lambda_3} j_y^{\lambda_3\lambda_2} j_y^{\lambda_2\lambda_1} j_y^{\lambda_1\lambda_5}}{4(\hbar\omega + i\eta) + \epsilon^{\lambda_1}_{\mathbf{k},\tau,s} - \epsilon^{\lambda_5}_{\mathbf{k},\tau,s}}$$
$$\left\{ \frac{1}{3(\hbar\omega + i\eta) + \epsilon^{\lambda_1}_{\mathbf{k},\tau,s} - \epsilon^{\lambda_4}_{\mathbf{k},\tau,s}} \right.$$
$$\times \left[\frac{U_{\lambda_1\lambda_2} - U_{\lambda_2\lambda_3}}{2(\hbar\omega + i\eta) + \epsilon^{\lambda_1}_{\mathbf{k},\tau,s} - \epsilon^{\lambda_3}_{\mathbf{k},\tau,s}} - \frac{U_{\lambda_2\lambda_3} - U_{\lambda_3\lambda_4}}{2(\hbar\omega + i\eta) + \epsilon^{\lambda_2}_{\mathbf{k},\tau,s} - \epsilon^{\lambda_4}_{\mathbf{k},\tau_s}} \right]$$
$$- \frac{1}{3(\hbar\omega + i\eta) + \epsilon^{\lambda_2}_{\mathbf{k},\tau,s} - \epsilon^{\lambda_5}_{\mathbf{k},\tau,s}}$$
$$\left. \times \left[\frac{U_{\lambda_2\lambda_3} - U_{\lambda_3\lambda_4}}{2(\hbar\omega + i\eta) + \epsilon^{\lambda_2}_{\mathbf{k},\tau,s} - \epsilon^{\lambda_4}_{\mathbf{k},\tau,s}} - \frac{U_{\lambda_3\lambda_4} - U_{\lambda_4\lambda_5}}{2(\hbar\omega + i\eta) + \epsilon^{\lambda_3}_{\mathbf{k},\tau,s} - \epsilon^{\lambda_5}_{\mathbf{k},\tau,s}} \right] \right\} . \tag{33}$$

For simplicity, we introduce the quantity $U_{\lambda\lambda'}$ as follows:

$$U_{\lambda\lambda'}(\mathbf{k},\omega,\tau,s) \equiv \frac{1}{\mathcal{S}} \frac{n_{\mathrm{F}}(\epsilon^{\lambda}_{\mathbf{k},\tau,s}) - n_{\mathrm{F}}(\epsilon^{\lambda'}_{\mathbf{k},\tau,s})}{\hbar\omega + \epsilon^{\lambda}_{\mathbf{k},\tau,s} - \epsilon^{\lambda'}_{\mathbf{k},\tau,s} + i\eta} , \tag{34}$$

where $\mathcal{S}$ is the sample area. All sums over band indices are limited to one conduction and one valance band, i.e. $\lambda, \lambda' = \mathrm{c}, \mathrm{v}$, and

$$n_{\mathrm{F}}(E) = \left\{ \exp\left(\frac{E-\mu}{k_{\mathrm{B}}T} \right) + 1 \right\}^{-1} \tag{35}$$

is the Fermi-Dirac distribution function at finite temperature T and chemical potential μ. In Supplementary Equations (30)-(33) we dropped the explicit functional dependence on $\mathbf{k}, \tau, s$, e.g. $j_y^{mn} = j_y^{mn}(\mathbf{k},\tau,s)$ and $U_{mn} = U_{mn}(\mathbf{k},\omega,\tau,s)$. We find most convenient to first carry out the sum over the band indices λ_i and then carry out numerically the integral over the wave vector $\mathbf{k}$.

The paramagnetic contributions to the even-order response functions, $\Pi^{(2),\mathrm{P}}_{yyy}$ and $\Pi^{(4),\mathrm{P}}_{yyyyy}$, vanish identically because $\epsilon^{\mathrm{c(v)}}_{\mathbf{k},\tau,s}$ is an even function of k_y. This property of the energy dispersion is protected by symmetry, and stems from time-reversal ($\mathcal{T}$) and reflection (σ_{v}) symmetries.

A microscopic calculation of even-order response functions requires the knowledge of diamagnetic contributions. These can be included with the aid of correlation functions involving the $\hat{\kappa}_{yy}$ operator. In fact, $\hat{\xi}_{yyy}$ could also contribute to diamagnetic responses. However, in our low-energy model $\hat{\xi}_{yyy}$ is identically zero. Similar to the paramagnetic case, $\hat{\kappa}_{yy}$ and $\hat{\xi}_{yyy}$ indicate the second-quantized form of the diamagnetic current operators (i.e. κ_{yy} and ξ_{yyy}). Diamagnetic contributions to the second- and third-order response functions are reported in Supplementary Figure 3, in terms of Feynman diagrams. For the sake of simplicity, we have not calculated diamagnetic contributions to the fourth-order response.

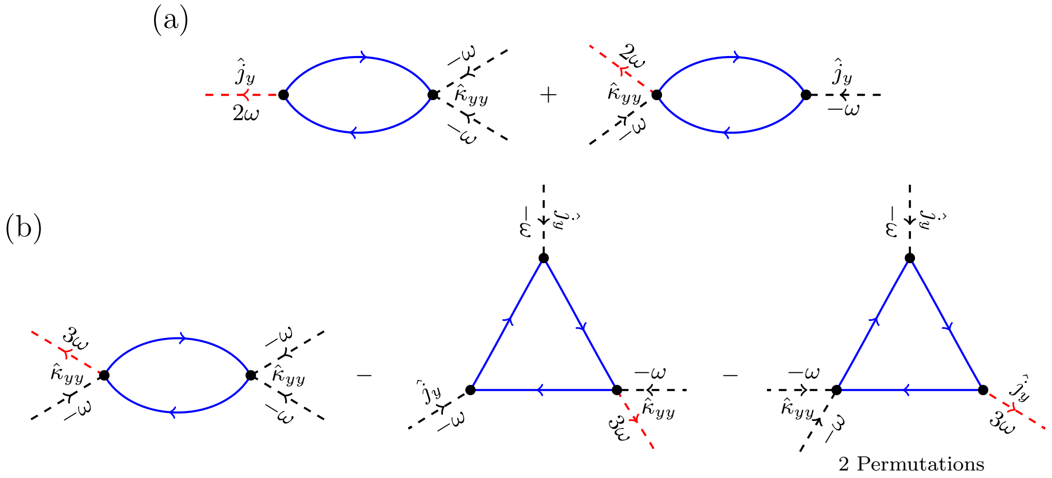

Supplementary Figure 3: Feynman diagrams for the *diamagnetic* contributions to the second- and third-order response functions. a) second-order response. b) third-order response.

According to Supplementary Figure 3a, the diamagnetic contribution to the second-order response is given by:

$$\Pi^{(2),\mathrm{D}}_{yyy}(-2\omega;\omega,\omega) = -\sum_{\mathbf{k},\tau,s}\sum_{\{\lambda_i\}}\left[U_{\lambda_1\lambda_2} j_y^{\lambda_1\lambda_2}\kappa_{yy}^{\lambda_2\lambda_1} + \widetilde{U}_{\lambda_1\lambda_2}\kappa_{yy}^{\lambda_1\lambda_2} j_y^{\lambda_2\lambda_1}\right] \ . \quad (36)$$

Similarly, the diamagnetic contribution to the third-order response, Supple-

mentary Figure 3b,is given by:

$$\Pi^{(3),\mathrm{D}}_{yyyy}(-3\omega;\omega,\omega,\omega) = \sum_{\mathbf{k},\tau,s}\sum_{\{\lambda_i\}} \widetilde{U}_{\lambda_1\lambda_2}\kappa^{\lambda_1\lambda_2}_{yy}\kappa^{\lambda_2\lambda_1}_{yy}$$
$$- \sum_{\mathbf{k},\tau,s}\sum_{\{\lambda_i\}} \frac{j^{\lambda_3\lambda_2}_{y} j^{\lambda_2\lambda_1}_{y}\kappa^{\lambda_1\lambda_3}_{yy}}{2(\hbar\omega+i\eta)+\epsilon^{\lambda_1}_{\mathbf{k},\tau,s}-\epsilon^{\lambda_3}_{\mathbf{k},\tau,s}}\left(U_{\lambda_1\lambda_2}-U_{\lambda_2\lambda_3}\right)$$
$$- \sum_{\mathbf{k},\tau,s}\sum_{\{\lambda_i\}}\sum_{\mathcal{P}}{}' \frac{\kappa^{\lambda_3\lambda_2}_{yy} j^{\lambda_2\lambda_1}_{y} j^{\lambda_1\lambda_3}_{y}}{3(\hbar\omega+i\eta)+\epsilon^{\lambda_1}_{\mathbf{k},\tau,s}-\epsilon^{\lambda_3}_{\mathbf{k},\tau,s}}\left(\widetilde{U}_{\lambda_1\lambda_2}-U_{\lambda_2\lambda_3}\right) . \tag{37}$$

Here, $\widetilde{U}_{\lambda_1\lambda_2} = U_{\lambda_1\lambda_2}(\mathbf{k},2\omega,\tau,s)$ with $\kappa^{mn}_{yy} = \kappa^{mn}_{yy}(\mathbf{k},\tau,s)$ is the matrix element of κ_{yy}.

Since our low-energy model is valid for a limited range of values of the wave vector $\mathbf{k}$, we must introduce an ultra-violet cut-off, which breaks gauge invariance[14]. We therefore need to regularize our final results to avoid un-physical response function. This can be accomplished[14] by considering the following gauge-regularized response tensors: $\Pi^{(n)}_{\ell i_1 i_2\ldots i_n} \equiv \Pi^{(n)}_{\ell i_1 i_2\ldots i_n} - \Pi^{(n)}_{\ell i_1 i_2\ldots i_n}\big|_{\{\omega_i\}\to 0}$.

We note that the summands in Supplementary Equations (31),(33) and (36) contain an *odd* number of matrix elements of the paramagnetic (j_y) and diamagnetic (κ_{yy}) current operators. In the absence of trigonal warping, the overall form-factor, which is proportional to these matrix elements, is an odd function of k_y: we therefore conclude that, *in the absence of trigonal warping*, $\Pi^{(2)}_{yyy}(-2\omega;\omega,\omega) = \Pi^{(4)}_{yyyyy}(-4\omega;\omega,\omega,\omega,\omega) = 0$. An identical conclusion was reached for other isotropic low-energy continuum model Hamiltonians, such as those describing gapped graphene[15] and biased 2LG[16, 17]. We therefore expect the second-order nonlinear response function $\Pi^{(2)}_{yyy}$ to be small compared to the third-order one, since it is controlled by a small trigonal warping correction ($\mathcal{H}_{\rm tw}$) in comparison with the fully isotropic leading term ($\mathcal{H}_{\rm i}$) in the low-energy model Hamiltonian. Of course, this conclusion is valid within the single-particle picture and in the low-energy limit, which we have relied on so far.

We now discuss how to evaluate the paramagnetic third order response function defined by the square diagram and given in Supplementary Equation (32). Similar steps are used for the diamagnetic part of the third order response, see Supplementary Figure 3b, as well as for the second and fourth order response functions. After performing the summation over the band

indices in Supplementary Equation (32), we obtain:

$$\Pi^{(3),\mathrm{P}}_{yyyy}(-3\omega;\omega,\omega,\omega) = (ev)^4 \int_0^{k_\mathrm{c}} \frac{kdk}{2\pi} \sum_{\tau,s} \sum_{n=1}^{3} \zeta_n(k,\tau,s) \frac{n_\mathrm{F}(\epsilon^\mathrm{c}_{\mathbf{k},\tau,s}) - n_\mathrm{F}(\epsilon^\mathrm{v}_{\mathbf{k},\tau,s})}{d_\mathrm{cv}(k,\tau,s)^2} \times \left[\frac{1}{d_\mathrm{cv}(k,\tau,s) + (\hbar\omega + i\eta)n} + \frac{1}{d_\mathrm{cv}(k,\tau,s) - (\hbar\omega + i\eta)n}\right] \tag{38}$$

where k_c is the ultra-violet cut-off, $v = t_0 a_0/\hbar$, $d_\mathrm{cv}(k,\tau,s) = \epsilon^\mathrm{c}_{\mathbf{k},\tau,s} - \epsilon^\mathrm{v}_{\mathbf{k},\tau,s}$ and $\zeta_n(k,\tau,s)$ are dimensionless functions given by:

$$\zeta_1(k,\tau,s) = \frac{1}{(ev)^4} \int_0^{2\pi} \frac{d\phi}{2\pi} \left[\frac{1}{6}(j_y^\mathrm{cc} - j_y^\mathrm{vv})^2 |j_y^\mathrm{cv}|^2 + \frac{1}{4}|j_y^\mathrm{cv}|^4\right] , \tag{39}$$

$$\zeta_2(k,\tau,s) = \frac{1}{(ev)^4} \int_0^{2\pi} \frac{d\phi}{2\pi} \left[-\frac{8}{3}(j_y^\mathrm{cc} - j_y^\mathrm{vv})^2 |j_y^\mathrm{cv}|^2\right] , \tag{40}$$

$$\zeta_3(k,\tau,s) = \frac{1}{(ev)^4} \int_0^{2\pi} \frac{d\phi}{2\pi} \left[\frac{9}{2}(j_y^\mathrm{cc} - j_y^\mathrm{vv})^2 |j_y^\mathrm{cv}|^2 - \frac{9}{4}|j_y^\mathrm{cv}|^4\right] , \tag{41}$$

where ϕ is the azimuthal angle of $\mathbf{k}$ vector. We consider the full anisotropic dispersion of 1L-MoS_2 and the integrations are handled numerically. Supplementary Equation (38) is also valid for other two-band LMs.

Using the isotropic model of 1L-MoS_2, see Supplementary Equation (1), we check the scaling of paramagnetic THG efficiency, $\Upsilon^\mathrm{P}_\mathrm{THG}$, with inter-band coupling, v. We consider the chemical potential inside the band gap (undoped $\mu = 0$) which implies $n_\mathrm{F}(\epsilon^\mathrm{c}_{\mathbf{k},\tau,s}) = 0$ and $n_\mathrm{F}(\epsilon^\mathrm{v}_{\mathbf{k},\tau,s}) = 1$ at zero temperature. Therefore we can rewrite Eq.38 in terms of density of states notation, $\rho_{\tau s}(\epsilon)$,

$$\Pi^{(3),\mathrm{P}}_{yyyy}(-3\omega;\omega,\omega,\omega) \approx -(ev)^4 \sum_{\tau,s} \sum_{n=1}^{3} \int_{-W}^{W} d\epsilon \frac{\rho_{\tau,s}(\epsilon)}{\epsilon^2} \zeta_n(\epsilon,\tau,s) \times \left[\frac{1}{\epsilon + (\hbar\omega + i\eta)n} + \frac{1}{\epsilon - (\hbar\omega + i\eta)n}\right] . \tag{42}$$

where W is the ultra-violet cut-off energy. Considering the isotropic $\boldsymbol{k}\cdot\boldsymbol{p}$ Hamiltonian for 1L-MoS_2, see Supplementary Equation (1), one can find the density of states for given spin and valley:

$$\rho_{\tau,s}(\epsilon) \approx \frac{|\epsilon|}{2\pi(\hbar v)^2} \left[\Theta\left(\epsilon - \epsilon^+_{\tau,s}\right) + \Theta\left(-\epsilon + \epsilon^-_{\tau,s}\right)\right] . \tag{43}$$

where $\epsilon^{\pm}_{\tau,s} = \pm[\Delta \pm (\lambda - \lambda_0)\tau s]/2$ stands for the conduction(+)/valence(−) band edge energy for each pair of spin and valley. For simplicity, we neglect α and β terms in the density of states. From Supplementary Equations (42,43), we get $\Pi^{(3),\mathrm{P}}_{yyyy} \propto v^2$. Therefore the paramagnetic THG efficiency scales like: $\Upsilon^{\mathrm{P}}_{\mathrm{THG}} \propto |\Pi^{(3),\mathrm{P}}_{yyyy}|^2 \propto v^4$. As discussed in the main text, this scaling is the main reason for having intense THG in 1L-MoS_2, because the inter-band coupling, v, is very strong, $v \approx 0.65c/300$ with c as the speed of light in vacuum.

Supplementary Note 4 Relative magnitude of nonlinear responses: ratios of irradiances

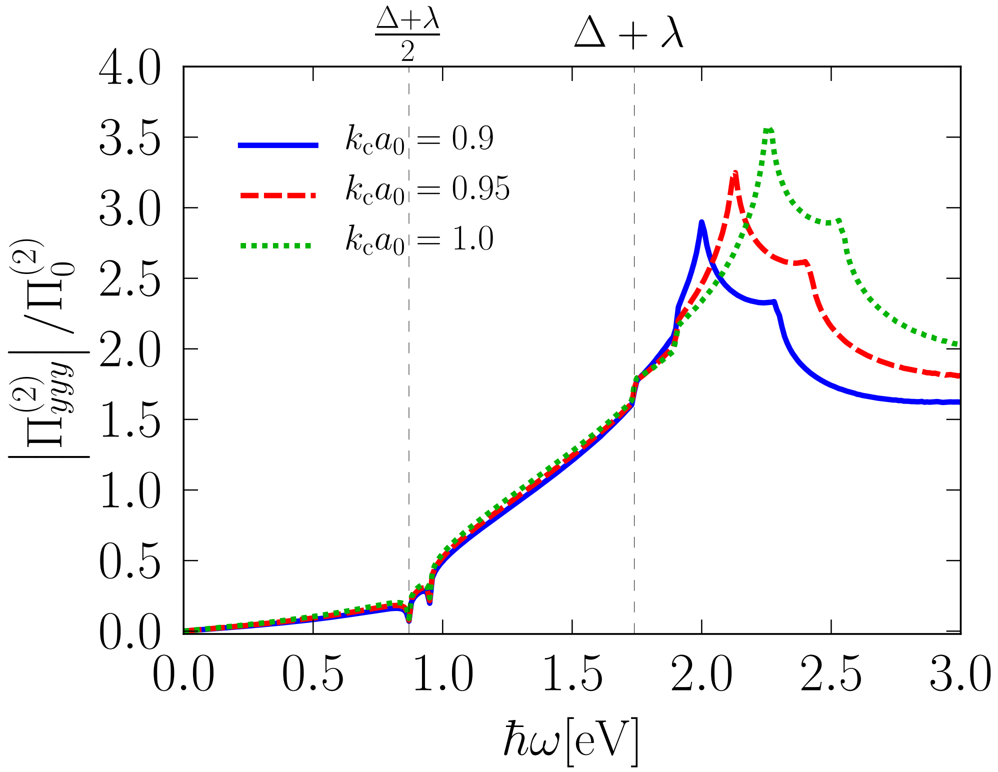

Supplementary Figure 4: Frequency dependence of the second-order response function $\Pi^{(2)}_{yyy}$ (in units of $\Pi^{(2)}_0$). Different curves refer to different values of the parameter k_c.

To quantify the *relative* magnitude of nonlinear harmonic signals, we calculate ratios between induced polarizations $P^{(n)}_y$ at different orders n in perturbation theory. For a linearly-polarized incident laser (e.g. $\mathbf{E} = |\mathbf{E}|\hat{\mathbf{y}}$) we

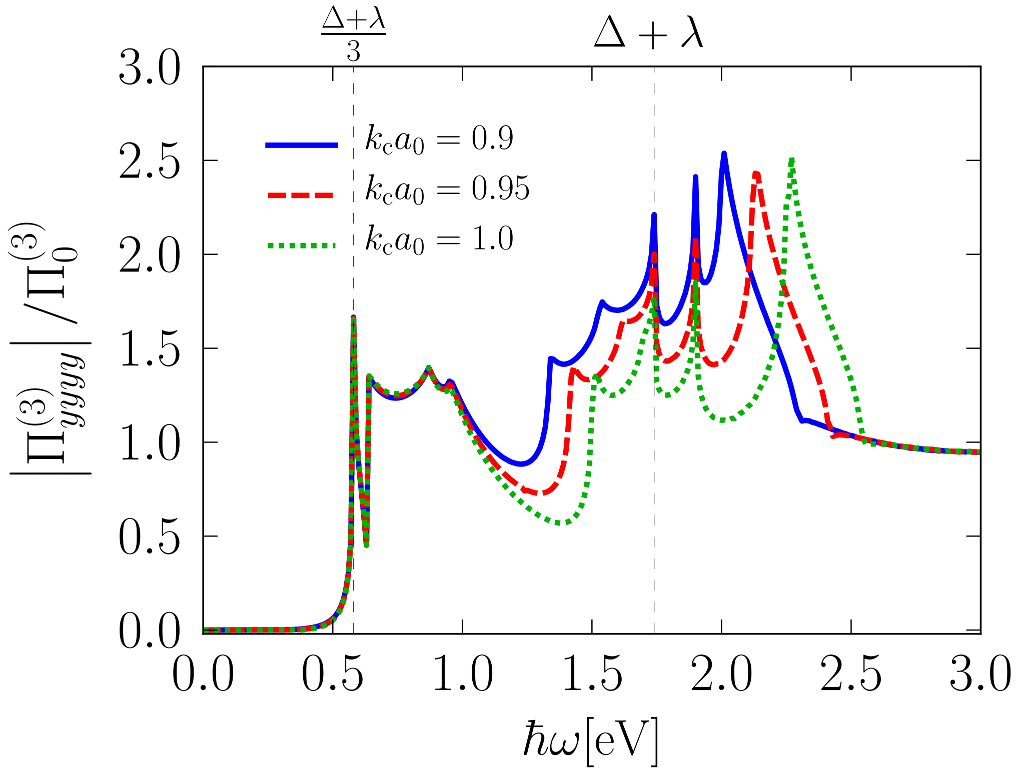

Supplementary Figure 5: Same as in Supplementary Figure 4, but for the case of the third-order response function.

find:

$$\left|\frac{P_y^{(n+1)}}{P_y^{(n)}}\right| = \left|\frac{\chi^{(n+1)}_{\underbrace{y\ldots y}_{n+2 \text{ times}}}|\mathbf{E}|}{\chi^{(n)}_{\underbrace{y\ldots y}_{n+1 \text{ times}}}}\right| = \left|\frac{\Pi^{(n+1)}_{\underbrace{y\ldots y}_{n+2 \text{ times}}}/\Pi_0^{(n+1)}}{(\hbar\omega + i\eta)/(1\mathrm{eV}) \times \Pi^{(n)}_{\underbrace{y\ldots y}_{n+1 \text{ times}}}/\Pi_0^{(n)}}\right|$$
$$\times \left(\frac{n\Pi_0^{(n+1)}\hbar}{(n+1)\Pi_0^{(n)}(1\mathrm{eV})}\right) \times |\mathbf{E}|$$
$$= \frac{n}{n+1} \times \frac{t_0}{1\mathrm{eV}} \times \frac{a_0}{1\mathrm{m}} \times \frac{|\mathbf{E}|}{1\mathrm{Vm}^{-1}} \times X_{n+1,n}(\omega) \; , \tag{44}$$

where

$$\Pi_0^{(n)} \equiv \frac{(et_0a_0/\hbar)^{n+1}}{8\pi a_0^2(1\mathrm{eV})^n} = \frac{(1\mathrm{eV})(1\mathrm{m}^{n-1})}{8\pi}\left(\frac{t_0}{1\mathrm{eV}}\right)^{n+1}\left(\frac{a_0}{1\mathrm{m}}\right)^{n-1}\left(\frac{e}{\hbar}\right)^{n+1} \tag{45}$$

and the quantities t_0 and a_0 have been introduced in the Hamiltonian $\mathcal{H}$. Π_0 represents the physical dimensions of the nonlinear current correlator

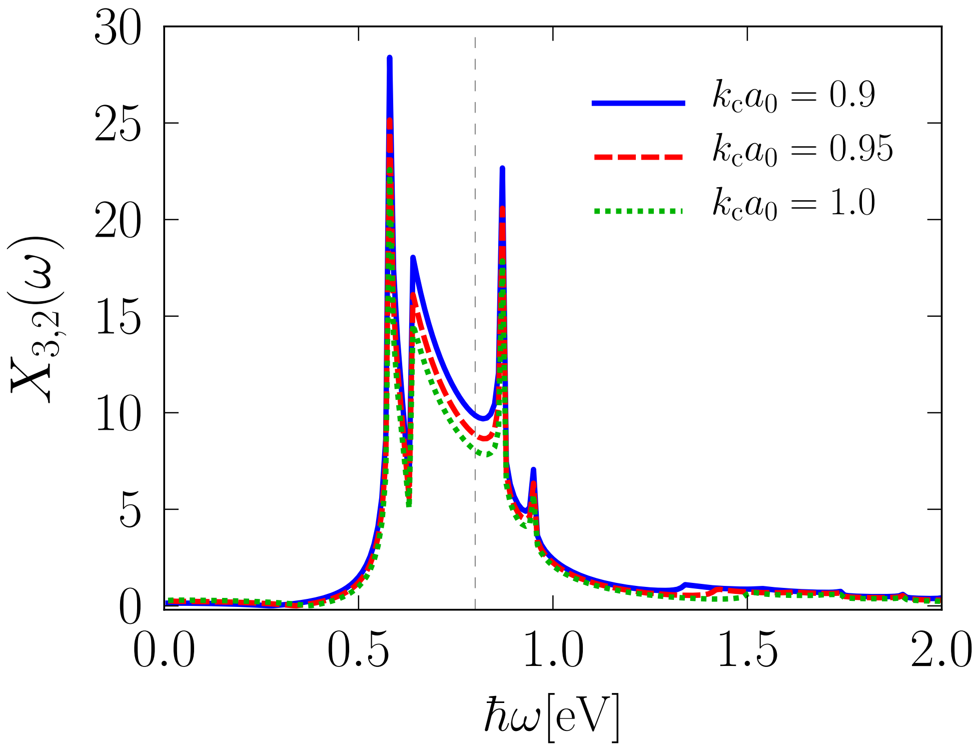

Supplementary Figure 6: Results for the $X_{3,2}$ as function of the pump laser frequency. Vertical dashed lines is positioned at $\hbar\omega = 0.8$ eV.

$\Pi^{(n)}_{\ell i_1 i_2 \ldots i_n}(-\omega_\Sigma; \omega_1, \omega_2, \ldots, \omega_n)$. The units of $\Pi^{(n)}_0$ are $\mathrm{C\,m}^{n-1}\mathrm{V}^{-n}\mathrm{s}^{-(n+1)}$. The dimensionless quantities $X_{n+1,n}$ are given by:

$$X_{n+1,n}(\omega) = \left| \frac{\Pi^{(n+1)}_{\underbrace{y\ldots y}_{n+2 \text{ times}}} / \Pi^{(n+1)}_0}{(\hbar\omega + i\eta)/(1\text{eV}) \times \Pi^{(n)}_{\underbrace{y\ldots y}_{n+1 \text{ times}}} / \Pi^{(n)}_0} \right| . \quad (46)$$

The amplitude of the electric field ($|\mathbf{E}|$) in Supplementary Equation (44) can be replaced by the power of the pump laser (P_{pump}) by using the following relation:

$$\frac{P_{\text{pump}}}{\pi(D/2)^2} = \frac{1}{2} n_r c \epsilon_0 |\mathbf{E}|^2 , \quad (47)$$

where $D \approx 1.85$ μm is the experimental spot size diameter, $n_r \approx 1$ is the refractive index of air, $c \approx 3 \times 10^8$ m s^{-1} is the speed of light in vacuum, and $\epsilon_0 \approx 8.85 \times 10^{-12}$ CV^{-1}m^{-1} is the vacuum electrical permittivity. Using

Maxwell's equations, we can obtain the following wave equation in a nonlinear medium[8]:

$$\nabla^2 \mathbf{E}^{(n)} + \left(\frac{\omega_n}{c}\right)^2 \boldsymbol{\epsilon}^{(1)}(\omega_n) \cdot \mathbf{E}^{(n)} = -\frac{1}{\epsilon_0}\left(\frac{\omega_n}{c}\right)^2 \mathbf{P}^{(n)} \ . \tag{48}$$

where $n = 2, 3, \ldots$ indicates the order of nonlinearity, $\boldsymbol{\epsilon}^{(1)}$ is the linear dielectric tensor and $\mathbf{P}^{(n)}$ is the n-th order polarization vector. The intensity $I^{(n)}$ of the n-th order nonlinear signal is proportional to the square of the induced electric field amplitude $E^{(n)} \propto \omega_n^2 P_y^{(n)}$ where $\omega_n = n\omega$ for the harmonic generation case. Inserting Supplementary Equation (47) in Supplementary Equation (44) we find:

$$\frac{I^{(n+1)}}{I^{(n)}} = \left(\frac{n+1}{n}\right)^2 \left|\frac{P_y^{(n+1)}}{P_y^{(n)}}\right|^2 = R_{n+1,n}(\omega) P_{\rm pump} \ , \tag{49}$$

where $R_{n+1,n}(\omega)$ in units of W^{-1} is given by:

$$R_{n+1,n}(\omega) = \frac{8[\mathrm{m\ s^{-1}}][\mathrm{CV^{-1}m^{-1}}]}{\pi n_r c \epsilon_0} \left[\frac{t_0/(1\mathrm{eV}) \times a_0/(1\mathrm{m})}{D/(1\mathrm{m})}\right]^2 [X_{n+1,n}(\omega)]^2 \ . \tag{50}$$

If we assume that the spot size of different harmonic-generated signals on the detector are equal to each other, we can write the following relation between power and intensity ratios:

$$\frac{I^{(n+1)}}{I^{(n)}} \approx \frac{P_{(n+1)\omega}}{P_{n\omega}} \ , \tag{51}$$

where $P_{n\omega}$ denotes the signal power of the n-th harmonic-generated signal.

Our main results for the 1L-MoS_2 nonlinear response functions are summarized in Supplementary Figures 4-6. We use the following values: $\Delta = 1.82$ eV, $\lambda_0 = 69$ meV, $\lambda = -80$ meV, $t_0 = 2.34$ eV, $\alpha = -0.01$, $\beta = -1.54$, $t_1 = -0.14$ eV, $t_2 = 1$ eV, $\alpha' = 0.44$, and $\beta' = -0.53$. These parameters are obtained from a tight-binding fitting[1] of LDA-DFT band structure calculations[19, 20]. In all our numerical results, we use $T = 300$ K and $\mu = 0$. In Supplementary Figures 4-6, we check the dependence of our results on the value of the ultra-violet cut-off, $k_{\rm c} \propto 1/a_0$. Note that $a_0 = a/\sqrt{3}$ with $a \approx 3.16$ Å is the lattice constant of 1L-MoS_2.

According to Supplementary Figures 4,5, the nonlinear response functions start to grow when $\hbar\omega$ is larger than $(\Delta + \lambda)/2$ and $(\Delta + \lambda)/3$ for the SHG and THG cases, respectively. $\Delta + \lambda$ is the optical band gap of MoS_2. In our energy range (< 1 eV) the spectra of the second and third order response functions are not very sensitive to the value of $k_{\rm c}$. The theoretical results in Fig.4c) of the main text are obtained by using Supplementary Equations (50),(49) for $\hbar\omega = 0.8$ eV.

Supplementary References

[1] Rostami, H., Roldán, R., Cappelluti, E., Asgari, R. & Guinea, F. Theory of strain in single-layer transition metal dichalcogenides. *Phys. Rev. B* **92**, 195402 (2015).

[2] Rostami, H., Asgari, R. & Guinea, F. Edge modes in zigzag and armchair ribbons of monolayer MoS_2. *J. Phys.: Condens. Matter* **28**, 495001 (2016).

[3] Zahid, F., Liu, L., Zhu, Y., Wang, J. & Guo, H. A generic tight-binding model for monolayer, bilayer and bulk MoS_2. *AIP Advances* **3**, 052111 (2013).

[4] Kormányos, A. et al. Monolayer MoS_2: trigonal warping, the Γ valley, and spin-orbit coupling effects. *Phys. Rev. B* **88**, 045416 (2013).

[5] Rostami, H., Moghaddam, A. G. & Asgari, R. Effective lattice Hamiltonian for monolayer MoS_2: Tailoring electronic structure with perpendicular electric and magnetic fields. *Phys. Rev. B* **88**, 085440 (2013).

[6] Alidoust, N. et al. Observation of monolayer valence band spin-orbit effect and induced quantum well states in MoX_2. *Nat. Comm.*. **5** 4673 (2014).

[7] Giuliani, G. F. & Vignale, G. *Quantum Theory of the Electron Liquid* (Cambridge University Press, 2005).

[8] Boyd, R.W. *Nonlinear Optics*, (Academic Press, 2003).

[9] Griffiths, D. J., *Introduction to Electrodynamics* (Pearson, 2012).

[10] Mahan, G. D. *Many-Particle Physics: Physics of Solids and Liquids* (Springer US, 1981).

[11] Butcher, P. N. & Cotter, D. *The elements of nonlinear optics* (Cambridge University Press, 1990).

[12] Jafari, S. A. Nonlinear optical response in gapped graphene. *J. Phys.: Condens. Matter* **24**, 205802 (2012).

[13] Rostami, H. & Polini, M. Theory of third harmonic generation in graphene: a diagrammatic approach. *Phys.Rev. B* **93**, 161411(R) (2016).

[14] Chirolli, L., Polini, M., Giovannetti, V. & MacDonald, A. H. Drude weight, cyclotron resonance, and the Dicke model of graphene cavity QED. *Phys. Rev. Lett.* **109**, 267404 (2012).

[15] Margulis, Vl. A., Muryumin, E. E. & Gaiduk, E. A. Optical second-harmonic generation from two-dimensional hexagonal crystals with broken space inversion symmetry. *J. Phys.: Condens. Matter* **25**, 195302 (2013).

[16] Wu, S., Mao, L., Jones, A. M., Yao, W., Zhang, C. & Xu, X. Quantum-enhanced tunable second-order optical nonlinearity in bilayer graphene. *Nano Lett.* **12**, 2032 (2012).

[17] Brun, S. J., & Pedersen, T. G. Intense and tunable second-harmonic generation in biased bilayer graphene. *Phys. Rev. B* **91**, 205405 (2015).

[18] Grüning, M. & Attaccalite, C. Second harmonic generation in h-BN and MoS_2 monolayers: Role of electron-hole interaction *Phys. Rev. B* **89**, 081102(R) (2014).

[19] Cappelluti, E., Roldán, R., Silva-Guillén, J. A., Ordejón, P. & Guinea, F. Tight-binding model and direct-gap/indirect-gap transition in single-layer and multilayer MoS_2. *Phys. Rev. B* **88**, 075409 (2013).

[20] Roldán, R. et al. Momentum dependence of spin-orbit interaction effects in single-layer and multi-layer transition metal dichalcogenides. *2D Mater.* **1**, 034003 (2014).
